# Crystal structures of four indole derivatives with a phenyl substituent at the 2-position and a carbonyl group at the 3-position: the *C*(6) N—H⋯O chain remains the same, but the weak reinforcing inter­actions are different

**DOI:** 10.1107/S2056989016002620

**Published:** 2016-02-20

**Authors:** Jamie R. Kerr, Laurent Trembleau, John M. D. Storey, James L. Wardell, William T. A. Harrison

**Affiliations:** a Department of Chemistry, University of Aberdeen, Meston Walk, Aberdeen AB24 3UE, Scotland; b Fundação Oswaldo Cruz, Instituto de Tecnologia em Fármacos-Far Manguinhos, 21041-250 Rio de Janeiro, RJ, Brazil

**Keywords:** crystal structure, indole, N—H⋯O hydrogen bond, *C*(6) chain, weak inter­actions

## Abstract

We describe the crystal structures of four indole derivatives with a phenyl ring at the 2-position and different carbonyl-linked substituents at the 3-position, namely 1-(2-phenyl-1*H*-indol-3-yl)ethanone, C_16_H_13_NO, (I), 2-cyclo­hexyl-1-(2-phenyl-1*H*-indol-3-yl)ethanone, C_22_H_23_NO, (II), 3,3-dimethyl-1-(2-phenyl-1*H*-indol-3-yl)butan-1-one, C_20_H_21_NO, (III), and 3-benzoyl-2-phenyl-1*H*-indole, C_21_H_15_NO, (IV). In each case, the carbonyl-group O atom lies close to the indole-ring plane and points towards the benzene ring. The dihedral angles between the indole ring system and 2-phenyl ring for these structures are clustered in a narrow range around 65°. The dominant inter­molecular inter­action in each case is an N—H⋯O hydrogen bond, which generates a *C*(6) chain, although each structure possesses a different crystal symmetry. The *C*(6) chains are consolidated by different (C—H⋯O, C—H⋯π and π–π stacking) weak inter­actions, with little consistency between the structures.

## Abstract 2

Four related indole derivatives crystallize with a consistent *C*(6) N—H⋯O chain motif, but in each case the reinforcing inter­actions and crystal symmetries are different.

## Chemical context   

Indole derivatives are widely studied due to their utility in many areas, including in the dye, plastics, agriculture and perfumery fields and as vitamin supplements and flavour enhancers (Barden, 2011 ▸). However, it is in the pharmaceutical field that most inter­est has been shown. Indoles, both naturally occurring and man-made, have been found to have activity as anti­hypertensive drugs, anti­depressants, anti­psychotic agents, anti-emetics, analgesics, anti-asthmatics, anti­virals, beta blockers, inhibitors of RNA polymerase-11, agonists for the cannabinoid receptor, non-nucleoside reverse transcriptase inhibitors, opioid agonists, sexual dysfunctional agents, *etc*. (França *et al.*, 2014 ▸; Kaushik *et al.*, 2013 ▸; Biswal *et al.*, 2012 ▸; Sharma *et al.*, 2010 ▸).
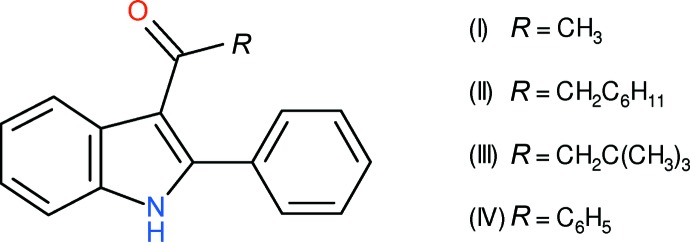


As part of our ongoing synthetic and biological (Kerr, 2013 ▸) and structural studies in this area (Kerr *et al.*, 2015 ▸) we report herein the crystal structures of four indole derivatives, namely: 1-(2-phenyl-1*H*-indol-3-yl)ethanone, C_16_H_13_NO, (I), 2-cyclo­hexyl-1-(2-phenyl-1*H*-indol-3-yl)ethanone, C_22_H_23_NO, (II), 3,3-dimethyl-1-(2-phenyl-1*H*-indol-3-yl)butan-1-one, C_20_H_21_NO, (III), and 3-benzoyl-2-phenyl-1*H*-indole, C_21_H_15_NO, (IV).

As we discuss below, each structure features *C*(6) N—H⋯O hydrogen-bonded chains but with different crystal symmetries and weak reinforcing effects (C—H⋯O and C—H⋯π inter­actions and aromatic π–π stacking).

## Structural commentary   

**Figure 1:**
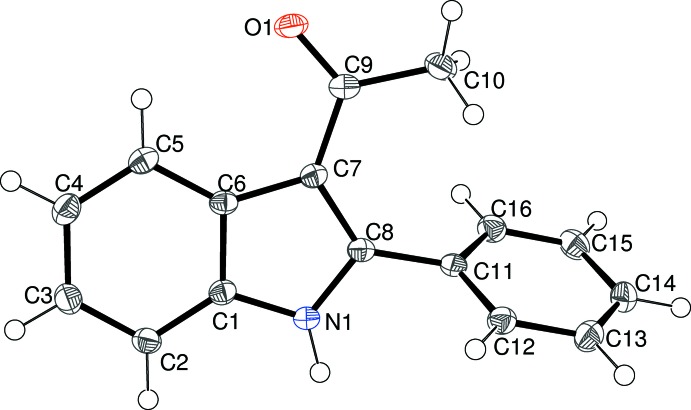
The mol­ecular structure of (I), showing 50% displacement ellipsoids.

The mol­ecular structure of (I) is illustrated in Fig. 1 ▸. The dihedral angles between the mean plane of the indole ring system (r.m.s. deviation = 0.018 Å) and the C9/C10/O1 grouping and the C11-benzene ring are 8.35 (4) and 65.44 (4)°, respectively. The C6—C7—C9 and C8—C7—C9 bond angles are 124.57 (9) and 129.04 (10)°, respectively. O1 is *syn* to H5 [C6—C7—C9—O1 = −8.14 (16)°] and a short intra­molecular contact occurs (H5⋯O1 = 2.54 Å), although we do not regard this as a bond. The C8—C7—C9—C10 torsion angle of −6.53 (16)° shows that C8 and C10 are almost eclipsed.

**Figure 2:**
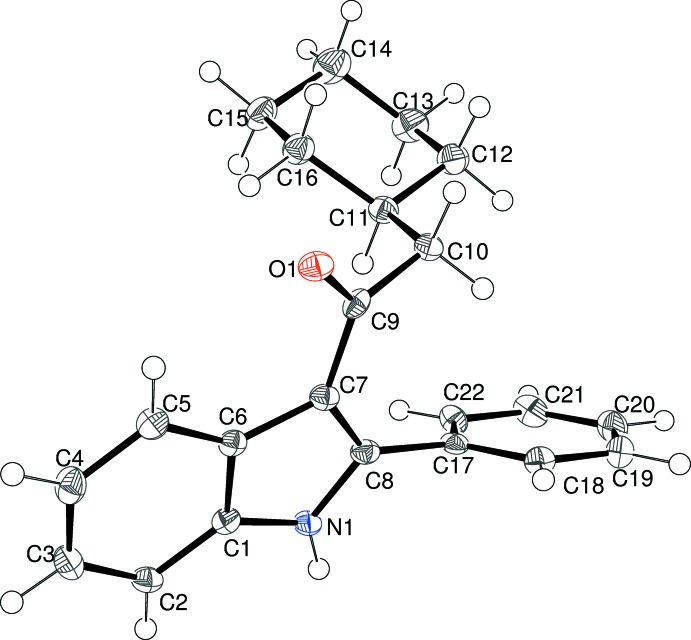
The mol­ecular structure of (II), showing 50% displacement ellipsoids.

The mol­ecular structure of (II) is shown in Fig. 2 ▸. The cyclo­hexyl ring adopts a normal chair conformation with the exocyclic C—C bond in an equatorial orientation. The dihedral angles between the indole ring system (r.m.s. deviation = 0.012 Å) and the C9/C10/O1 grouping and the C11-benzene ring are 21.17 (14) and 68.58 (8)°, respectively. The C6—C7—C9 and C8—C7—C9 bond angles are 124.3 (2) and 129.3 (2)°, respectively and the C8—C7—C9—C10 torsion angle is 16.2 (4)°. This is significantly larger than the equivalent value for (I), possibly due to steric inter­actions between the pendant ring systems: the twist about the C7—C9 bond in (II) is in the opposite sense to that in (I) [C6—C7—C9—O1 = 16.4 (3)°].

**Figure 3:**
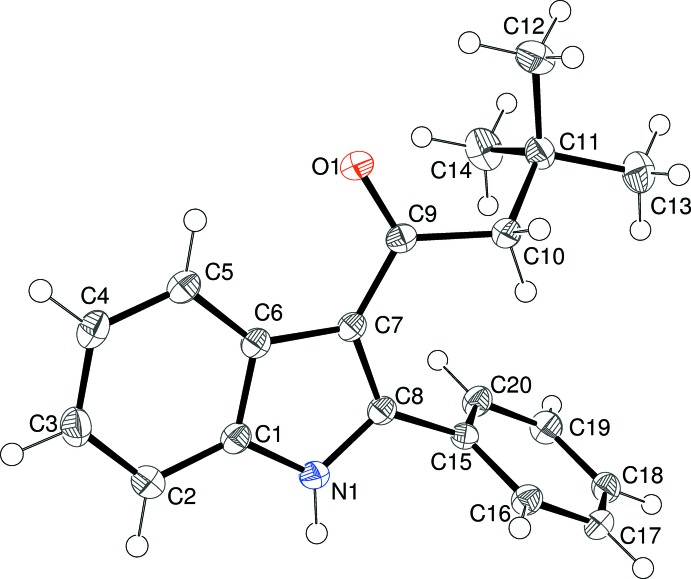
The mol­ecular structure of (III), showing 50% displacement ellipsoids.

Fig. 3 ▸ shows the mol­ecular structure of (III). The indole ring system (r.m.s. deviation = 0.007 Å) subtends dihedral angles of 15.60 (8) and 70.07 (3)° with the C9/C10/O1 grouping and the C15 benzene ring, respectively. The C7—C9—C10—C11 torsion angle is 137.54 (9)°. and the C6—C7—C9 and C8—C7—C9 bond angles are 124.3 (2) and 129.3 (2)°, respectively. The C8—C7—C9—C10 torsion angle is −14.06 (15)°. The C6—C7—C9—O1 torsion angle of −13.96 (14)° shows that the C=O bond is slightly twisted away from the indole plane.

**Figure 4:**
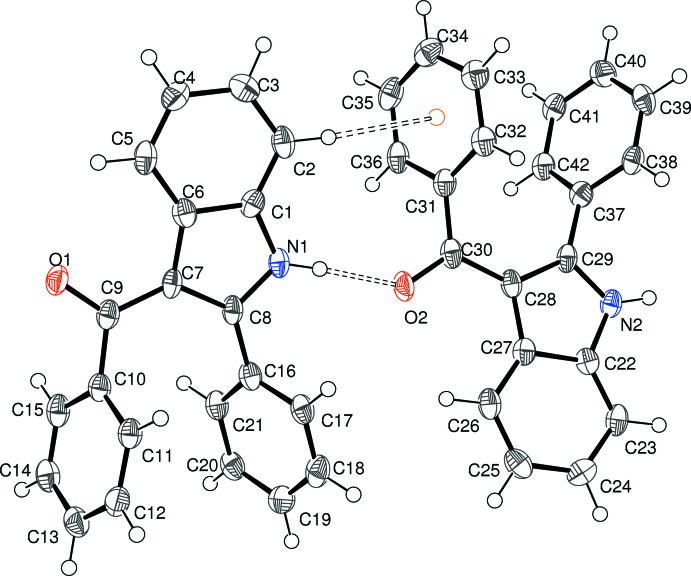
The mol­ecular structure of (IV), showing 50% displacement ellipsoids. The N—H⋯O and C—H⋯π bonds are indicated by double-dashed lines.

Compound (IV) crystallizes with two mol­ecules in the asymmetric unit, as shown in Fig. 4 ▸. The mol­ecules have similar but not identical conformations, as indicated by the r.m.s. overlay fit of 0.102 Å for the 23 non-hydrogen atoms. The main differences are a slightly different twist of the benzene ring at the 2-position and the fact that atoms C10 and C31 deviate slightly from the indole ring plane, but in opposite directions. This is reflected in the metrical data for the individual mol­ecules: in the N1-species, the indole ring system (r.m.s. deviation = 0.009 Å) subtends dihedral angles of 7.32 (15), 64.66 (7), and 54.57 (7)° with the C9/C10/O1 group, the C10-ring and the C16-ring, respectively. Equivalent data for the N2-mol­ecule (r.m.s. deviation for the indole ring system = 0.009 Å) are 9.76 (13) (C30/C31/O2), 60.92 (7) (C31-ring) and 56.97 (7)° (C37-ring). In the N1-mol­ecule, the C6—C7—C9 and C8—C7—C9 bond angles are 123.5 (2) and 130.5 (2)°, respectively and the C8—C7—C9—C10 torsion angle is 7.1 (4)°. Equivalent data for the N2-mol­ecule are C27—C28—C30 [124.0 (2)°], C29—C28—C30 [130.2 (3)°] and C29—C28—C30—C31 [–9.7 (4)°].

## Supra­molecular features   

In each structure, as might be expected, the dominant supra­molecular motif is an N—H⋯O=C hydrogen bond, which generates a *C*(6) chain in every case. However, it is notable that the same chain motif is reinforced by different weak inter­actions in these structures, as described below and listed in Tables 1 ▸–4 ▸ ▸ ▸, for (I)–(IV), respectively.

**Figure 5:**
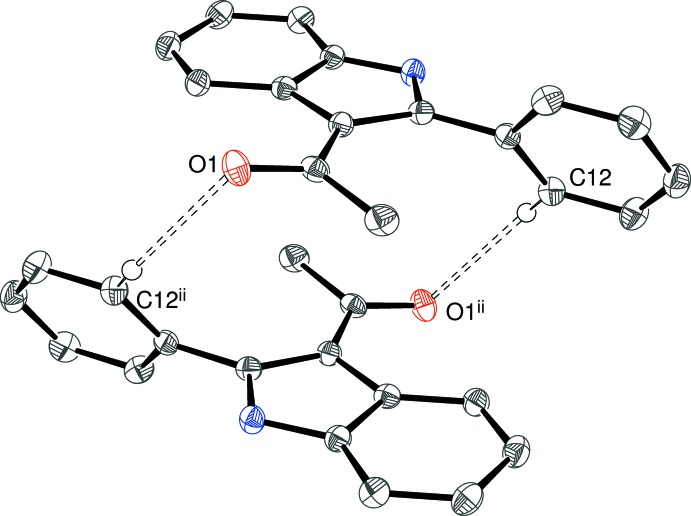
An inversion dimer in the crystal of (I) linked by a pair of C—H⋯O inter­actions (double-dashed lines). Symmetry code as in Table 1 ▸.

**Figure 6:**
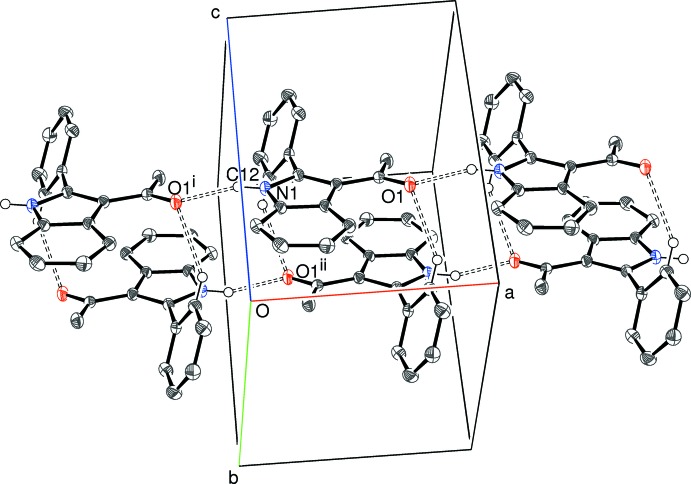
Partial packing diagram for (I), showing the formation of [100] double chains linked by N—H⋯O and C—H⋯O hydrogen bonds (double-dashed lines). Symmetry codes as in Table 1 ▸.

In the triclinic crystal of (I), the N1—H1⋯O1^i^ [symmetry code: (i) *x* – 1, *y*, *z*] hydrogen bond links the mol­ecules into [100] chains with the aforementioned *C*(6) chain motif in which adjacent mol­ecules are related by translational symmetry. In addition, a C12—H12⋯O1^ii^ [symmetry code: (ii) 1 – *x*, 1 – *y*, 1 – *z*] inter­action is seen. By itself, this generates inversion dimers (Fig. 5 ▸) with an 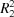
(14) motif: the twisting of the C11 ring relative to the indole skeleton appears to optimize the geometry for this inter­action. Taken together, the N—H⋯O and C—H⋯O bonds in (I) lead to double chains propagating in [100] (Fig. 6 ▸). Inversion symmetry means that the sense of the N—H⋯O bonds are opposed in the two chains. Packing between the chains does not feature any directional inter­actions beyond typical van der Waals contacts and there is no aromatic π–π stacking in (I).

**Figure 7:**
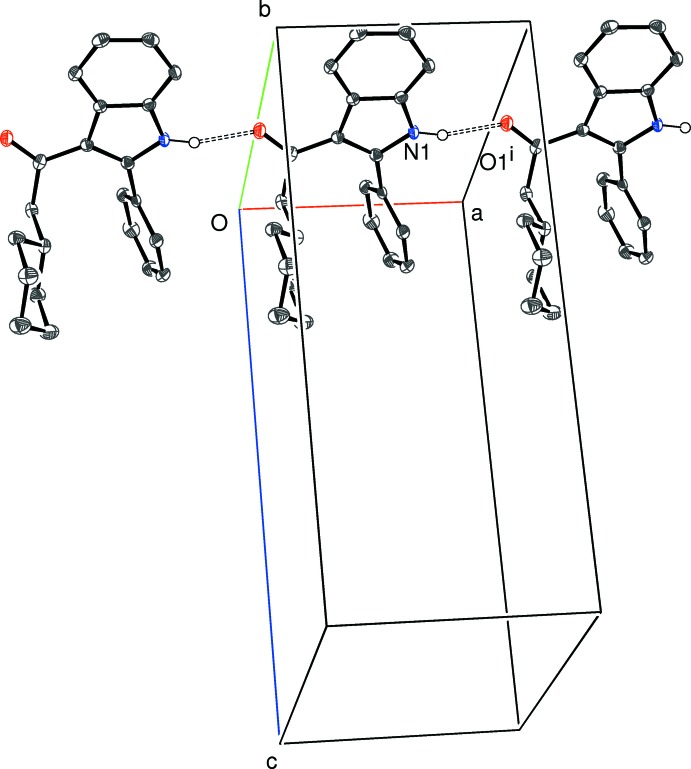
Partial packing diagram for (II), showing the formation of [100] chains linked by N—H⋯O hydrogen bonds (double-dashed lines). Symmetry code as in Table 2 ▸.

**Figure 8:**
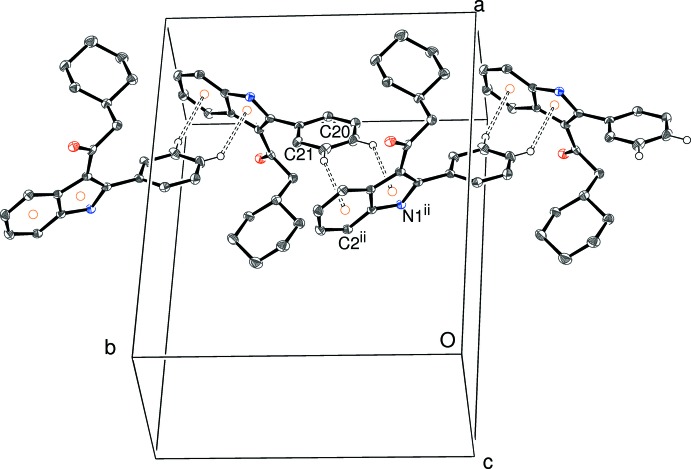
Partial packing diagram for (II) showing the formation of [010] chains linked by pairs of C—H⋯π inter­actions. Symmetry code as in Table 2 ▸.

In the ortho­rhom­bic crystal of (II), the mol­ecules are linked by N1—H1—O2^i^ [symmetry code: (i) *x* + 1, *y*, *z*] hydrogen bonds into [100] chains (Fig. 7 ▸) characterized by a *C*(6) motif: adjacent mol­ecules are again related by simple unit-cell translation. There is no reinforcement of the chain bonding in this case, but a pair of weak C—H⋯π inter­actions occur, which arise from adjacent C—H groupings of the pendant C17–C22 benzene ring to an adjacent indole ring (Fig. 8 ▸), and result in [010] chains. Taken together, the N—H⋯O and C—H⋯π bonds in (II) lead to (001) sheets.

**Figure 9:**
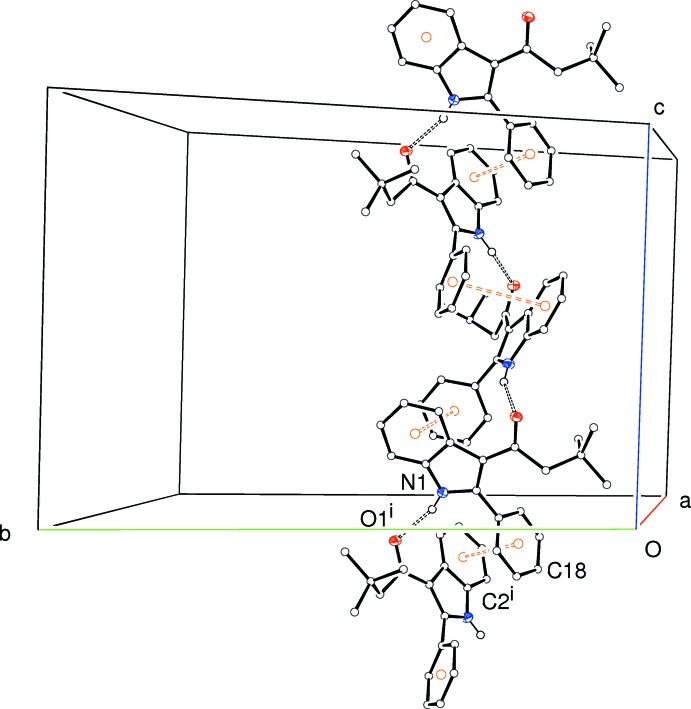
Partial packing diagram for (III), showing the formation of [001] chains linked by N—H⋯O hydrogen bonds (double-dashed lines) and reinforced by aromatic π–π stacking contacts. Symmetry code as in Table 3 ▸.

The extended structure in (III) conforms to rhombohedral (trigonal) crystal symmetry. Once again, adjacent mol­ecules are linked into *C*(6) chains by N1—H1⋯O2^i^ [symmetry code: (i) 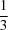 − *x* + *y*, 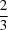 − *x*, *z* − 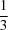] and symmetry-equivalent hydrogen bonds. The chain propagates in the [001] direction (Fig. 9 ▸) and the chain that incorporates the asymmetric mol­ecule describes an anti­clockwise helix, when viewed from above, about the 3_1_ symmetry element at (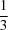, 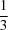, *z*). The centrosymmetric space group leads, of course, to an equal number of clockwise and anti­clockwise helices in the crystal. The chains are reinforced by aromatic π–π stacking between the pendant C15–C20 ring and the C1–C6 ring of the indole system with the same symmetry relation as the N—H⋯O hydrogen bond: the centroid separation is 3.7565 (8) Å and the inter-plane angle is 0.00 (6)°]. There appears to be no directional inter­actions between the chains beyond van der Waals contacts.

**Figure 10:**
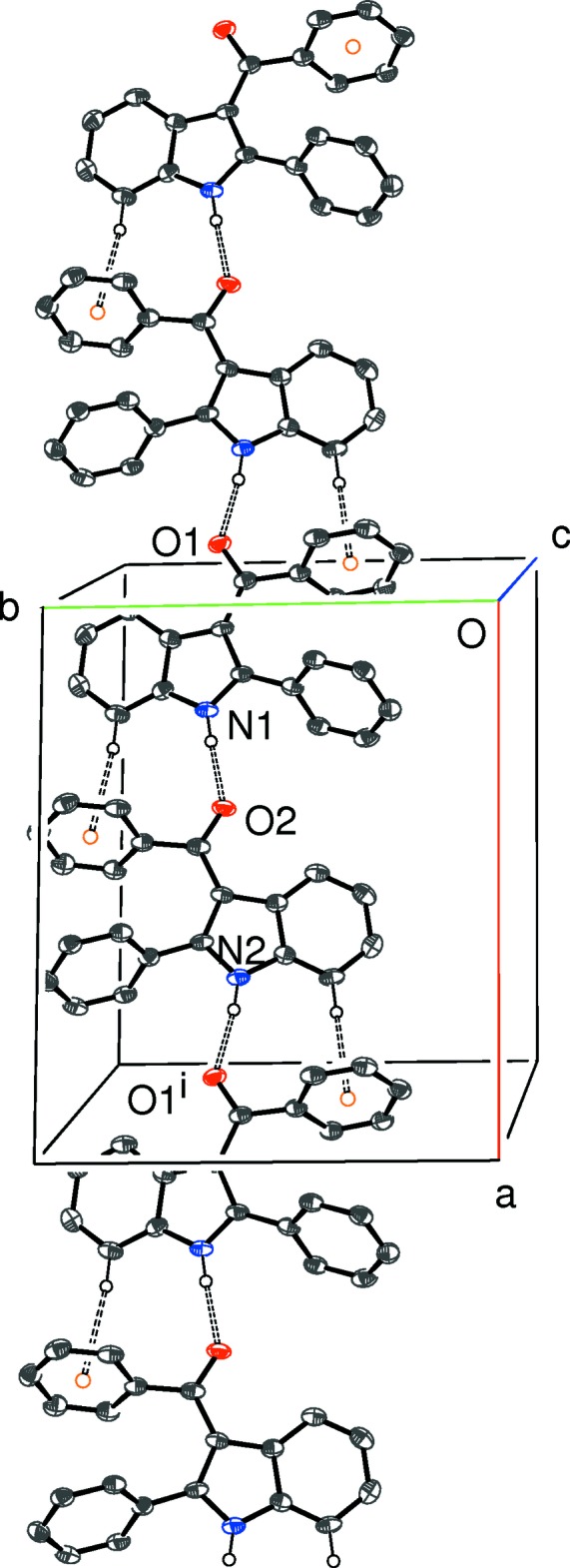
Partial packing diagram for (IV), showing the formation of [100] chains of alternating A and B mol­ecules linked by N—H⋯O hydrogen bonds (double-dashed lines) and reinforced by aromatic π–π stacking contacts. Symmetry code as in Table 4 ▸.

Compound (IV) crystallizes in a monoclinic space group. The *C*(6) chain motif (Fig. 10 ▸) is built up from alternating N1- and N2-mol­ecules, with simple translation in the [100] direction generating the chain from the starting pair. In this case, the chain is consolidated by C—H⋯π inter­actions (involving both the N1 and N2 mol­ecules) with the donor C—H group lying *syn* (*i.e*., C2—H2*A* and C23—H23, compare Fig. 4 ▸) to the N—H group in the indole ring system and the acceptor ring being the pendant phenyl group attached to the carbonyl group at the 3-position of the ring system (*i.e*., the C10 and C31 rings). Adjacent N1- and N2-mol­ecules in the chain are ‘flipped’ by approximately 180° with respect to each other, so the chain has approximate local 2_1_ symmetry. The packing for (IV) also features two C—H⋯O and three inter-chain C—H⋯π inter­actions, which generate a three-dimensional network.

## Database survey   

A search of the Cambridge Structural Database (Groom & Allen, 2014 ▸) for indole derivatives with a phenyl substituent at the 2-position and a carbonyl group at the 3-position yielded five hits, namely: 3,5-dimethyl 2-(3,4-di­meth­oxy­phen­yl)indole-3,5-di­carboxyl­ate di­chloro­methane solvate (refcode GUXMUI; Hwu *et al.*, 2009 ▸), 2-(3-*t*-butyl­dimethyl­sil­oxy-4-meth­oxy­phen­yl)-3-(3,4,5-tri­meth­oxy­benzo­yl)-6-meth­oxy­indole (IFIDEG; Hadimani *et al.*, 2002 ▸), 1-(2-(2-meth­oxy­phen­yl)-1*H*-indol-3-yl)ethanone (MEYYOG; Coffman *et al.*, 2013 ▸), (5-methyl-2-(4-methyl­phen­yl)-1*H*-indol-3-yl)(phen­yl)methanone (MOLDIC; Shi *et al.*, 2014 ▸) and 1-(6-methyl-2-phenyl-1*H*-indol-3-yl)ethanone (SUHWUP; Huang *et al.*, 2014 ▸). All of these structures feature *C*(6) chains linked by N—H⋯O hydrogen bonds, as seen in the compounds described here, which we may thus conclude is a consistent supra­molecular motif in these phases.

## Synthesis and crystallization   

To prepare (I), 2-phenyl­indole (2.129 g, 11.0 mmol) was suspended in dry di­chloro­methane (45 ml) at 273 K and a 1.0 *M* solution of Et_2_AlCl in hexa­nes (16.5 ml, 16.5 mmol) was added slowly with stirring. A solution of benzoyl chloride (1.919 ml, 16.5 mmol) in dry di­chloro­methane (20 ml) was then added dropwise and the mixture was stirred at 273 K for a further 2 h. Water (30 ml) was added to quench the reaction then the solution was poured into 1.0 *M* HCl(aq) (100 ml) and the organic layer collected after shaking. The organic solution was washed with water (30 ml, twice) and saturated NaCl(aq) (30 ml) then dried over sodium sulfate, filtered and reduced under vacuum. Flash chromatography (1:4 EtOAc, hexa­nes) afforded 1-(2-phenyl-1*H*-indol-3-yl)ethanone as a colourless solid (2.257 g, 69%). Colourless slabs of (I) were recrystallized from ethanol solution at room temperature. δC(101 MHz; DMSO-*d*_6_) 192.6 (Cq), 144.5 (Cq), 140.3 (Cq), 136.3 (CH), 132.0 (CH), 131.8 (Cq), 130.0 (CH), 129.5(CH), 128.9 (CH), 128.6 (Cq), 128.5 (Cq), 128.2 (CH), 123.3 (CH), 121.8 (CH), 121.0 (CH), 112.6 (CH) and 112.3 (Cq); δH(400 MHz; DMSO-*d*_6_) 12.16 (1H, *br s*), 7.76 (1H, *d*, J 7.8), 7.71 (2H, *d*, *J* 8.4), 7.58–7.56 (3H, m), 7.49 (2H, *t*, *J* 6.9), 7.38–7.17 (4H, *m*), 7.13 (1H, *t*, *J* 7.2) and 7.09–7.04 (1H, *m*); *R_f_* 0.20 (1:4 EtOAc, hexa­nes); m.p. 495–496 K; IR (KBr, cm^−1^) 3393, 3060, 2968, 1707, 1551, 1208, 1116, 891 and 745; HRMS (ESI) for C_21_H_16_NO [*M* + H]^+^ calculated 298.1233, found 298.1230.

To prepare (II), a suspension of 2-phenyl­indole (567 mg, 2.93 mmol) in dry di­chloro­methane (20 ml) was cooled to 273 K over ice–water before the dropwise addition of a 1.0 *M* solution of Et_2_AlCl in hexane (4.4 ml, 4.40 mmol). After stirring for 30 min, a solution of cyclo­hexyl­acetyl chloride (675 ml, 4.40 mmol) in dry di­chloro­methane (20 ml) was added dropwise and stirring was resumed over ice–water for 2 h. Water (50 ml) was added slowly and after warming to room temperature, the mixture was added to a 1.0 *M* solution of HCl(aq) (50 ml). The organic phase was collected, washed with water (20 ml) and saturated NaCl(aq) (20 ml), dried (sodium sulfate), filtered and evaporated under reduced pressure. Flash chromatography (1:7 EtOAc, hexa­nes then 1:5 EtOAc,hexa­nes) gave 2-cyclo­hexyl-1-(2-phenyl-1*H*-indol-3-yl)ethanone as a yellow solid (92 mg, 10%). Colourless rods of (II) were recrystallized from ethanol solution at room temperature. δC(101 MHz; CDCl3) 198.4 (Cq), 143.5 (Cq), 135.1 (Cq), 132.9 (CH), 129.7 (CH), 129.5 (CH), 128.6 (Cq), 127.4 (Cq), 123.5 (CH), 122.5 (CH), 122.4 (CH), 115.8 (CH), 110.8 (Cq), 49.7 (CH_2_), 35.0 (CH_2_), 33.2 (CH), 26.2 (CH_2_) and 26.1 (CH_2_); δH(400 MHz; CDCl_3_) 8.51 (1H, *br s*), 8.27–8.25 (1H, *m*), 7.48–7.38 (5H, *m*), 7.32–7.28 (1H, *m*), 7.23–7.18 (2H, *m*), 2.30 (2H, *d*, *J* 6.8), 1.53–1.40 (5H, *m*), 1.19–0.93 (4H, *m*) and 0.66 (2H, *q*, *J* 10.7); *R_f_* 0.23 (1:5 EtOAc, hexa­nes); m.p. 447 K; IR (KBr, cm^−1^) 3197, 3023, 2857, 1715, 1567, 1411, 1215, 1154 and 763; HRMS (ESI) for C_22_H_24_NO [*M* + H]^+^ calculated 318.1859, found 318.1855.

To prepare (III), a 1.0 *M* solution of Et_2_AlCl in hexane (20 ml, 20 mmol) was added dropwise to a suspension of 2-phenyl­indole (2.536 g, 13.1 mmol) in dry di­chloro­methane (DCM) (56 ml) at 273 K. After 30 min stirring, a solution of 3,3-di­methyl­butanoyl chloride (2.75 ml, 19.8 mmol) in dry DCM (55 ml) was added slowly and stirring was resumed for 2 h. Water (30 ml) was added and the solution was shaken with 1.0 *M* HCl(aq) (30 ml). The organic phase was collected, washed with water (20 ml) and saturated NaCl(aq) (20 ml), dried (sodium sulfate), filtered and evaporated under vacuum. Flash chromatography (5:1 DCM, hexa­nes) yielded 3,3-dimethyl-1-(2-phenyl-1*H*-indol-3-yl)butan-1-one as a cream-coloured solid (1.909 g, 50%). Colourless blocks of (III) were recrystallized from ethanol solution at room temperature. δC(101 MHz; CDCl_3_) 199.1(Cq), 142.9 (Cq), 135.2 (Cq), 132.9 (CH), 129.7 (CH), 129.5 (CH), 128.8 (Cq), 127.4 (Cq), 123.6 (CH), 122.4 (CH), 122.3 (CH), 117.3 (CH), 110.7 (Cq), 53.8 (CH_2_), 31.9 (Cq) and 29.9 (CH_3_); δH(400 MHz; CDCl_3_) 8.37 (1H, *br s*), 8.23–8.21 (1H, *m*), 7.48–7.19 (8H, *m*), 2.34 (2H, *s*) and 0.77 (9H, *s*); *R_f_* 0.31 (5:1 DCM, hexa­nes); m.p. 441–443 K; IR (KBr, cm^−1^) 3186, 2998, 2954, 1710, 1454, 1411, 1202, 1150, 939 and 736; HRMS (ESI) for C_20_H_22_NO [*M* + H]^+^ calculated, 292.1702, found, 292.1697.

To prepare (IV), 2-phenyl­indole (2.129 g, 11.0 mmol) was suspended in dry DCM (45 ml) at 273 K and a 1.0 *M* solution of Et_2_AlCl in hexa­nes (16.5 ml, 16.5 mmol) was added slowly with stirring. A solution of benzoyl chloride (1.919 ml, 16.5 mmol) in dry DCM (20 ml) was then added dropwise and the mixture was stirred at 273 K for a further 2 h. Water (30 ml) was added to quench the reaction then the solution was poured into 1.0 *M* HCl(aq) (100 ml) and the organic layer collected after shaking. The DCM solution was washed with water (30 ml, twice) and saturated NaCl(aq) (30 ml) then dried (sodium sulfate), filtered and reduced under vacuum. Flash chromatography (1:4 EtOAc, hexa­nes) afforded 3-benzoyl-2-phenyl-1*H*-indole as a colourless solid (2.257 g, 69%). Colourless blocks and slabs of (IV) were recrystallized from ethanol solution at room temperature. δC(101 MHz; DMSO-*d*_6_) 192.6 (Cq), 144.5 (Cq), 140.3 (Cq), 136.3 (CH), 132.0 (CH), 131.8 (Cq), 130.0 (CH), 129.5 (CH), 128.9 (CH), 128.6 (Cq), 128.5 (Cq), 128.2 (CH), 123.3 (CH), 121.8 (CH), 121.0 (CH), 112.6 (CH) and 112.3 (Cq); δH(400 MHz; DMSO-*d*6) 12.16 (1H, *br s*), 7.76 (1H, *d*, *J* 7.8), 7.71 (2H, *d*, *J* 8.4), 7.58–7.56 (3H, *m*), 7.49 (2H, *t*, *J* 6.9), 7.38–7.17 (4H, *m*), 7.13 (1H, *t*, *J* 7.2) and 7.09–7.04 (1H, *m*); Rf 0.20 (1:4 EtOAc, hexa­nes); m.p. 495–496 K; IR (KBr, cm^−1^) 3393, 3060, 2968, 1707, 1551, 1208, 1116, 891 and 745; HRMS (ESI) for C_21_H_16_NO [*M* + H]^+^ calculated 298.1233, found 298.1230.

## Refinement   

Crystal data, data collection and structure refinement details for (I)–(IV) are summarized in Table 5 ▸. The N-bound H atoms were located in difference maps and their positions freely refined [for (IV) they were refined as riding atoms in their as-found relative positions]. The C-bound H atoms were geometrically placed (C—H = 0.93–0.98 Å) and refined as riding atoms. The constraint *U*_iso_(H) = 1.2*U*_eq_(carrier) or 1.5*U*_eq_(methyl carrier) was applied in all cases. The methyl H atoms (if any) were allowed to rotate, but not to tip, to best fit the electron density. Compound (II) crystallizes in space group *P*2_1_2_1_2_1_ but the absolute structure was indeterminate in the present experiment. The crystal of (III) was found to contain highly disordered solvent mol­ecules. Attempts to model the disorder were ineffective and the contribution to the scattering was removed with the SQUEEZE (Spek, 2015 ▸) option in *PLATON* (Spek, 2009 ▸), which revealed a solvent-accessible volume of 244.3 Å^3^ per unit cell and 19 ‘solvent’ electrons per unit cell. The stated formula, mol­ecular mass, density, etc. for (III) in Table 5 ▸ do not take the solvent into account.

## Supplementary Material

Crystal structure: contains datablock(s) I, II, III, IV, global. DOI: 10.1107/S2056989016002620/xu5883sup1.cif

Structure factors: contains datablock(s) I. DOI: 10.1107/S2056989016002620/xu5883Isup2.hkl

Structure factors: contains datablock(s) II. DOI: 10.1107/S2056989016002620/xu5883IIsup3.hkl

Structure factors: contains datablock(s) III. DOI: 10.1107/S2056989016002620/xu5883IIIsup4.hkl

Structure factors: contains datablock(s) IV. DOI: 10.1107/S2056989016002620/xu5883IVsup5.hkl

Supporting information file. DOI: 10.1107/S2056989016002620/xu5883Isup6.cml

Supporting information file. DOI: 10.1107/S2056989016002620/xu5883IIsup7.cml

Supporting information file. DOI: 10.1107/S2056989016002620/xu5883IIIsup8.cml

Supporting information file. DOI: 10.1107/S2056989016002620/xu5883IVsup9.cml

CCDC references: 1453285, 1453284, 1453283, 1453282

Additional supporting information:  crystallographic information; 3D view; checkCIF report

## Figures and Tables

**Table 1:** Hydrogen-bond geometry (Å, °) for (I)

*D*—H⋯*A*	*D*—H	H⋯*A*	*D*⋯*A*	*D*—H⋯*A*
N1—H1⋯O1^i^	0.898 (15)	2.018 (15)	2.8630 (12)	156.3 (12)
C12—H12⋯O1^ii^	0.95	2.53	3.3583 (14)	146

Symmetry codes: (i) 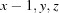; (ii) 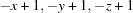.

**Table 2:** Hydrogen-bond geometry (Å, °) for (II) *Cg*1 and *Cg*2 are the centroids of the N1/C1/C6–C8 ring and the C1–C6 ring, respectively.

*D*—H⋯*A*	*D*—H	H⋯*A*	*D*⋯*A*	*D*—H⋯*A*
N1—H1⋯O1^i^	0.91 (3)	1.94 (3)	2.806 (3)	158 (2)
C20—H20⋯*Cg*1^ii^	0.95	2.75	3.503 (3)	136
C21—H21⋯*Cg*2^ii^	0.95	2.61	3.437 (3)	146

Symmetry codes: (i) 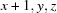; (ii) 
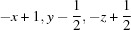
.

**Table 3:** Hydrogen-bond geometry (Å, °) for (III)

*D*—H⋯*A*	*D*—H	H⋯*A*	*D*⋯*A*	*D*—H⋯*A*
N1—H1⋯O1^i^	0.909 (13)	1.953 (13)	2.7950 (11)	153.3 (12)

Symmetry code: (i) 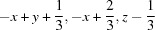.

**Table 4:** Hydrogen-bond geometry (Å, °) for (IV) *Cg*8, *Cg*1, *Cg*7, *Cg*3 and *Cg*6 are the centroids of the C31–C36, N1/C1/C6–C8, C22–C27, C10–C15 and N2/C22/C27–C29 rings, respectively.

*D*—H⋯*A*	*D*—H	H⋯*A*	*D*⋯*A*	*D*—H⋯*A*
N1—H1⋯O2	0.88	1.91	2.786 (3)	176
N2—H2⋯O1^i^	0.88	1.90	2.775 (3)	171
C20—H20⋯O1^ii^	0.95	2.44	3.324 (3)	155
C41—H41⋯O2^iii^	0.95	2.37	3.239 (3)	152
C2—H2*A*⋯*Cg*8	0.95	2.81	3.715 (3)	158
C14—H14⋯*Cg*1^ii^	0.95	2.89	3.616 (3)	134
C17—H17⋯*Cg*7^iv^	0.95	2.62	3.508 (3)	156
C23—H23⋯*Cg*3^i^	0.95	2.72	3.608 (3)	156
C35—H35⋯*Cg*6^iii^	0.95	2.80	3.527 (3)	134

Symmetry codes: (i) 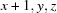; (ii) 
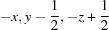
; (iii) 
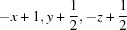
; (iv) 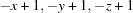.

**Table 5:** Experimental details

	(I)	(II)	(III)	(IV)
Crystal data				
Chemical formula	C_16_H_13_NO	C_22_H_23_NO	C_20_H_21_NO	C_21_H_15_NO
*M*_r_	235.27	317.41	291.38	297.34
Crystal system, space group	Triclinic, *P* 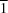	Orthorhombic, *P*2_1_2_1_2_1_	Trigonal, *R* 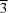	Monoclinic, *P*2_1_/*c*
Temperature (K)	100	100	100	100
*a*, *b*, *c* (Å)	7.4136 (5), 7.5070 (5), 10.9519 (8)	7.3587 (5), 13.225 (1), 17.5445 (13)	23.3305 (16), 23.3305 (16), 15.3681 (11)	14.5065 (10), 11.7911 (9), 18.6961 (13)
α, β, γ (°)	101.274 (7), 92.218 (6), 97.893 (7)	90, 90, 90	90, 90, 120	90, 107.782 (2), 90
*V* (Å^3^)	590.74 (7)	1707.4 (2)	7244.3 (9)	3045.1 (4)
*Z*	2	4	18	8
Radiation type	Mo *K*α	Mo *K*α	Mo *K*α	Mo *K*α
μ (mm^−1^)	0.08	0.08	0.07	0.08
Crystal size (mm)	0.40 × 0.14 × 0.05	0.60 × 0.16 × 0.14	0.66 × 0.60 × 0.24	0.22 × 0.03 × 0.01

Data collection				
Diffractometer	Rigaku Mercury CCD	Rigaku Mercury CCD	Rigaku Mercury CCD	Rigaku Mercury CCD
No. of measured, independent and observed [*I* > 2σ(*I*)] reflections	7753, 2703, 2432	8189, 3490, 2802	32188, 3690, 3070	20680, 6949, 4461
*R*_int_	0.033	0.045	0.037	0.063
(sin θ/λ)_max_ (Å^−1^)	0.650	0.650	0.649	0.649

Refinement				
*R*[*F* ^2^ > 2σ(*F* ^2^)], *wR*(*F* ^2^), *S*	0.040, 0.114, 1.07	0.051, 0.100, 1.21	0.036, 0.092, 1.08	0.076, 0.215, 1.05
No. of reflections	2703	3490	3690	6949
No. of parameters	167	221	205	415
H-atom treatment	H atoms treated by a mixture of independent and constrained refinement	H atoms treated by a mixture of independent and constrained refinement	H atoms treated by a mixture of independent and constrained refinement	H-atom parameters constrained
Δρ_max_, Δρ_min_ (e Å^−3^)	0.37, −0.19	0.23, −0.22	0.29, −0.18	0.58, −0.23

Computer programs: *CrystalClear* (Rigaku, 2012 ▸), *SHELXS97* and *SHELXL97* (Sheldrick, 2008 ▸), *ORTEP-3 for Windows* (Farrugia, 2012 ▸) and *publCIF* (Westrip, 2010 ▸).

## supplementary crystallographic information

### (I) 1-(2-Phenyl-1H-indol-3-yl)ethanone Crystal data

**Table:**

C_16_H_13_NO	*Z* = 2
*M**_r_* = 235.27	*F*(000) = 248
Triclinic, *P*1	*D*_x_ = 1.323 Mg m^−^^3^
*a* = 7.4136 (5) Å	Mo *K*α radiation, λ = 0.71075 Å
*b* = 7.5070 (5) Å	Cell parameters from 7537 reflections
*c* = 10.9519 (8) Å	θ = 2.8–27.5°
α = 101.274 (7)°	µ = 0.08 mm^−^^1^
β = 92.218 (6)°	*T* = 100 K
γ = 97.893 (7)°	Slab, colourless
*V* = 590.74 (7) Å^3^	0.40 × 0.14 × 0.05 mm

### (I) 1-(2-Phenyl-1H-indol-3-yl)ethanone Data collection

**Table:**

Rigaku Mercury CCD diffractometer	2432 reflections with *I* > 2σ(*I*)
Radiation source: fine-focus sealed tube	*R*_int_ = 0.033
Graphite monochromator	θ_max_ = 27.5°, θ_min_ = 2.8°
ω scans	*h* = −9→9
7753 measured reflections	*k* = −8→9
2703 independent reflections	*l* = −14→13

### (I) 1-(2-Phenyl-1H-indol-3-yl)ethanone Refinement

**Table:**

Refinement on *F*^2^	Primary atom site location: structure-invariant direct methods
Least-squares matrix: full	Secondary atom site location: difference Fourier map
*R*[*F*^2^ > 2σ(*F*^2^)] = 0.040	Hydrogen site location: inferred from neighbouring sites
*wR*(*F*^2^) = 0.114	H atoms treated by a mixture of independent and constrained refinement
*S* = 1.07	*w* = 1/[σ^2^(*F*_o_^2^) + (0.0655*P*)^2^ + 0.1376*P*] where *P* = (*F*_o_^2^ + 2*F*_c_^2^)/3
2703 reflections	(Δ/σ)_max_ = 0.001
167 parameters	Δρ_max_ = 0.37 e Å^−^^3^
0 restraints	Δρ_min_ = −0.19 e Å^−^^3^

### (I) 1-(2-Phenyl-1H-indol-3-yl)ethanone Special details

**Table:**

Geometry. All esds (except the esd in the dihedral angle between two l.s. planes) are estimated using the full covariance matrix. The cell esds are taken into account individually in the estimation of esds in distances, angles and torsion angles; correlations between esds in cell parameters are only used when they are defined by crystal symmetry. An approximate (isotropic) treatment of cell esds is used for estimating esds involving l.s. planes.
Refinement. Refinement of F^2^ against ALL reflections. The weighted R-factor wR and goodness of fit S are based on F^2^, conventional R-factors R are based on F, with F set to zero for negative F^2^. The threshold expression of F^2^ > 2sigma(F^2^) is used only for calculating R-factors(gt) etc. and is not relevant to the choice of reflections for refinement. R-factors based on F^2^ are statistically about twice as large as those based on F, and R- factors based on ALL data will be even larger.

### (I) 1-(2-Phenyl-1H-indol-3-yl)ethanone Fractional atomic coordinates and isotropic or equivalent isotropic displacement parameters (Å^2^)

**Table:**

	*x*	*y*	*z*	*U*_iso_*/*U*_eq_	
C1	0.17290 (14)	0.14842 (14)	0.39273 (10)	0.0172 (2)	
C2	0.05849 (15)	0.06773 (15)	0.28675 (10)	0.0202 (2)	
H2	−0.0705	0.0566	0.2886	0.024*	
C3	0.14074 (16)	0.00441 (15)	0.17853 (10)	0.0226 (2)	
H3	0.0672	−0.0521	0.1045	0.027*	
C4	0.33210 (16)	0.02277 (15)	0.17698 (10)	0.0226 (2)	
H4	0.3854	−0.0186	0.1011	0.027*	
C5	0.44441 (15)	0.09970 (14)	0.28344 (10)	0.0195 (2)	
H5	0.5733	0.1100	0.2811	0.023*	
C6	0.36453 (14)	0.16230 (14)	0.39494 (10)	0.0166 (2)	
C7	0.43386 (13)	0.25110 (14)	0.52057 (9)	0.0165 (2)	
C8	0.28168 (14)	0.28785 (14)	0.58734 (10)	0.0166 (2)	
C9	0.62677 (14)	0.29478 (14)	0.56241 (10)	0.0180 (2)	
C10	0.68827 (15)	0.41016 (16)	0.68934 (11)	0.0236 (2)	
H10A	0.8136	0.4713	0.6881	0.035*	
H10B	0.6838	0.3315	0.7512	0.035*	
H10C	0.6075	0.5028	0.7114	0.035*	
C11	0.26171 (13)	0.37691 (14)	0.71834 (10)	0.0173 (2)	
C12	0.18773 (15)	0.53985 (15)	0.74297 (10)	0.0202 (2)	
H12	0.1523	0.5941	0.6761	0.024*	
C13	0.16559 (16)	0.62334 (16)	0.86536 (11)	0.0250 (3)	
H13	0.1162	0.7352	0.8818	0.030*	
C14	0.21514 (16)	0.54433 (17)	0.96377 (10)	0.0245 (3)	
H14	0.1998	0.6020	1.0472	0.029*	
C15	0.28710 (16)	0.38114 (17)	0.93984 (11)	0.0247 (3)	
H15	0.3203	0.3262	1.0069	0.030*	
C16	0.31064 (15)	0.29786 (15)	0.81775 (10)	0.0221 (2)	
H16	0.3605	0.1862	0.8018	0.027*	
N1	0.12741 (12)	0.22642 (12)	0.51028 (8)	0.0180 (2)	
H1	0.011 (2)	0.2342 (19)	0.5287 (13)	0.022*	
O1	0.74295 (10)	0.23746 (12)	0.49276 (7)	0.0234 (2)	

### (I) 1-(2-Phenyl-1H-indol-3-yl)ethanone Atomic displacement parameters (Å^2^)

**Table:**

	*U*^11^	*U*^22^	*U*^33^	*U*^12^	*U*^13^	*U*^23^
C1	0.0171 (5)	0.0158 (5)	0.0194 (5)	0.0044 (4)	0.0017 (4)	0.0038 (4)
C2	0.0172 (5)	0.0212 (5)	0.0220 (5)	0.0045 (4)	−0.0013 (4)	0.0034 (4)
C3	0.0253 (6)	0.0220 (5)	0.0196 (5)	0.0059 (4)	−0.0027 (4)	0.0011 (4)
C4	0.0263 (6)	0.0214 (5)	0.0210 (5)	0.0080 (4)	0.0047 (4)	0.0026 (4)
C5	0.0187 (5)	0.0183 (5)	0.0228 (5)	0.0058 (4)	0.0043 (4)	0.0042 (4)
C6	0.0153 (5)	0.0148 (5)	0.0206 (5)	0.0036 (4)	0.0011 (4)	0.0048 (4)
C7	0.0152 (5)	0.0160 (5)	0.0190 (5)	0.0036 (4)	0.0021 (4)	0.0038 (4)
C8	0.0142 (5)	0.0157 (5)	0.0201 (5)	0.0026 (4)	0.0006 (4)	0.0040 (4)
C9	0.0152 (5)	0.0178 (5)	0.0226 (5)	0.0028 (4)	0.0020 (4)	0.0079 (4)
C10	0.0171 (5)	0.0259 (6)	0.0260 (6)	0.0003 (4)	−0.0027 (4)	0.0038 (4)
C11	0.0119 (4)	0.0192 (5)	0.0196 (5)	0.0004 (4)	0.0012 (4)	0.0025 (4)
C12	0.0195 (5)	0.0217 (5)	0.0201 (5)	0.0049 (4)	0.0011 (4)	0.0048 (4)
C13	0.0273 (6)	0.0244 (6)	0.0234 (6)	0.0089 (4)	0.0018 (4)	0.0018 (4)
C14	0.0239 (5)	0.0296 (6)	0.0180 (5)	0.0035 (5)	0.0014 (4)	0.0003 (4)
C15	0.0249 (6)	0.0287 (6)	0.0208 (5)	0.0027 (4)	−0.0032 (4)	0.0073 (4)
C16	0.0215 (5)	0.0205 (5)	0.0243 (5)	0.0049 (4)	−0.0017 (4)	0.0039 (4)
N1	0.0132 (4)	0.0215 (5)	0.0186 (4)	0.0035 (3)	0.0009 (3)	0.0020 (3)
O1	0.0144 (4)	0.0320 (5)	0.0256 (4)	0.0064 (3)	0.0041 (3)	0.0075 (3)

### (I) 1-(2-Phenyl-1H-indol-3-yl)ethanone Geometric parameters (Å, º)

**Table:**

C1—N1	1.3809 (13)	C9—C10	1.5041 (15)
C1—C2	1.3933 (15)	C10—H10A	0.9800
C1—C6	1.4090 (14)	C10—H10B	0.9800
C2—C3	1.3849 (15)	C10—H10C	0.9800
C2—H2	0.9500	C11—C12	1.3916 (15)
C3—C4	1.4075 (16)	C11—C16	1.3965 (15)
C3—H3	0.9500	C12—C13	1.3903 (15)
C4—C5	1.3841 (16)	C12—H12	0.9500
C4—H4	0.9500	C13—C14	1.3884 (16)
C5—C6	1.4038 (14)	C13—H13	0.9500
C5—H5	0.9500	C14—C15	1.3852 (17)
C6—C7	1.4471 (14)	C14—H14	0.9500
C7—C8	1.3979 (13)	C15—C16	1.3893 (16)
C7—C9	1.4576 (14)	C15—H15	0.9500
C8—N1	1.3643 (14)	C16—H16	0.9500
C8—C11	1.4819 (14)	N1—H1	0.898 (15)
C9—O1	1.2383 (13)		
			
N1—C1—C2	128.98 (10)	C9—C10—H10A	109.5
N1—C1—C6	107.83 (9)	C9—C10—H10B	109.5
C2—C1—C6	123.20 (10)	H10A—C10—H10B	109.5
C3—C2—C1	117.20 (10)	C9—C10—H10C	109.5
C3—C2—H2	121.4	H10A—C10—H10C	109.5
C1—C2—H2	121.4	H10B—C10—H10C	109.5
C2—C3—C4	120.75 (10)	C12—C11—C16	119.15 (10)
C2—C3—H3	119.6	C12—C11—C8	119.48 (9)
C4—C3—H3	119.6	C16—C11—C8	121.35 (10)
C5—C4—C3	121.56 (10)	C13—C12—C11	120.08 (10)
C5—C4—H4	119.2	C13—C12—H12	120.0
C3—C4—H4	119.2	C11—C12—H12	120.0
C4—C5—C6	118.86 (10)	C14—C13—C12	120.47 (11)
C4—C5—H5	120.6	C14—C13—H13	119.8
C6—C5—H5	120.6	C12—C13—H13	119.8
C5—C6—C1	118.36 (10)	C15—C14—C13	119.76 (10)
C5—C6—C7	134.81 (10)	C15—C14—H14	120.1
C1—C6—C7	106.77 (9)	C13—C14—H14	120.1
C8—C7—C6	106.36 (9)	C14—C15—C16	119.98 (10)
C8—C7—C9	129.04 (10)	C14—C15—H15	120.0
C6—C7—C9	124.57 (9)	C16—C15—H15	120.0
N1—C8—C7	109.13 (9)	C15—C16—C11	120.57 (10)
N1—C8—C11	118.22 (9)	C15—C16—H16	119.7
C7—C8—C11	132.65 (10)	C11—C16—H16	119.7
O1—C9—C7	119.96 (10)	C8—N1—C1	109.91 (9)
O1—C9—C10	119.00 (10)	C8—N1—H1	127.8 (9)
C7—C9—C10	121.05 (9)	C1—N1—H1	122.3 (9)
			
N1—C1—C2—C3	178.05 (10)	C6—C7—C9—O1	−8.14 (16)
C6—C1—C2—C3	−2.09 (16)	C8—C7—C9—C10	−6.53 (16)
C1—C2—C3—C4	−0.36 (16)	C6—C7—C9—C10	171.46 (10)
C2—C3—C4—C5	1.74 (17)	N1—C8—C11—C12	−63.28 (13)
C3—C4—C5—C6	−0.67 (16)	C7—C8—C11—C12	116.36 (13)
C4—C5—C6—C1	−1.67 (15)	N1—C8—C11—C16	114.80 (11)
C4—C5—C6—C7	−178.46 (11)	C7—C8—C11—C16	−65.56 (16)
N1—C1—C6—C5	−176.98 (9)	C16—C11—C12—C13	0.87 (16)
C2—C1—C6—C5	3.14 (16)	C8—C11—C12—C13	178.99 (10)
N1—C1—C6—C7	0.65 (11)	C11—C12—C13—C14	−0.65 (18)
C2—C1—C6—C7	−179.23 (9)	C12—C13—C14—C15	−0.02 (18)
C5—C6—C7—C8	176.50 (11)	C13—C14—C15—C16	0.46 (18)
C1—C6—C7—C8	−0.55 (11)	C14—C15—C16—C11	−0.23 (17)
C5—C6—C7—C9	−1.87 (18)	C12—C11—C16—C15	−0.43 (16)
C1—C6—C7—C9	−178.92 (9)	C8—C11—C16—C15	−178.52 (10)
C6—C7—C8—N1	0.26 (11)	C7—C8—N1—C1	0.15 (12)
C9—C7—C8—N1	178.53 (10)	C11—C8—N1—C1	179.87 (9)
C6—C7—C8—C11	−179.41 (11)	C2—C1—N1—C8	179.36 (10)
C9—C7—C8—C11	−1.14 (19)	C6—C1—N1—C8	−0.51 (12)
C8—C7—C9—O1	173.88 (10)		

### (I) 1-(2-Phenyl-1H-indol-3-yl)ethanone Hydrogen-bond geometry (Å, º)

**Table:**

*D*—H···*A*	*D*—H	H···*A*	*D*···*A*	*D*—H···*A*
N1—H1···O1^i^	0.898 (15)	2.018 (15)	2.8630 (12)	156.3 (12)
C12—H12···O1^ii^	0.95	2.53	3.3583 (14)	146

Symmetry codes: (i) *x*−1, *y*, *z*; (ii) −*x*+1, −*y*+1, −*z*+1.

### (II) 2-Cyclohexyl-1-(2-phenyl-1H-indol-3-yl)ethanone Crystal data

**Table:**

C_22_H_23_NO	*D*_x_ = 1.235 Mg m^−^^3^
*M**_r_* = 317.41	Mo *K*α radiation, λ = 0.71075 Å
Orthorhombic, *P*2_1_2_1_2_1_	Cell parameters from 4889 reflections
*a* = 7.3587 (5) Å	θ = 1.9–27.5°
*b* = 13.225 (1) Å	µ = 0.08 mm^−^^1^
*c* = 17.5445 (13) Å	*T* = 100 K
*V* = 1707.4 (2) Å^3^	Rod, colourless
*Z* = 4	0.60 × 0.16 × 0.14 mm
*F*(000) = 680	

### (II) 2-Cyclohexyl-1-(2-phenyl-1H-indol-3-yl)ethanone Data collection

**Table:**

Rigaku Mercury CCD diffractometer	2802 reflections with *I* > 2σ(*I*)
Radiation source: fine-focus sealed tube	*R*_int_ = 0.045
Graphite monochromator	θ_max_ = 27.5°, θ_min_ = 2.8°
ω scans	*h* = −9→9
8189 measured reflections	*k* = −17→17
3490 independent reflections	*l* = −22→18

### (II) 2-Cyclohexyl-1-(2-phenyl-1H-indol-3-yl)ethanone Refinement

**Table:**

Refinement on *F*^2^	Secondary atom site location: difference Fourier map
Least-squares matrix: full	Hydrogen site location: inferred from neighbouring sites
*R*[*F*^2^ > 2σ(*F*^2^)] = 0.051	H atoms treated by a mixture of independent and constrained refinement
*wR*(*F*^2^) = 0.100	*w* = 1/[σ^2^(*F*_o_^2^) + (0.0111*P*)^2^ + 0.987*P*] where *P* = (*F*_o_^2^ + 2*F*_c_^2^)/3
*S* = 1.21	(Δ/σ)_max_ < 0.001
3490 reflections	Δρ_max_ = 0.23 e Å^−^^3^
221 parameters	Δρ_min_ = −0.22 e Å^−^^3^
0 restraints	Extinction correction: *SHELXL*, Fc^*^=kFc[1+0.001xFc^2^λ^3^/sin(2θ)]^-1/4^
Primary atom site location: structure-invariant direct methods	Extinction coefficient: 0.0029 (5)

### (II) 2-Cyclohexyl-1-(2-phenyl-1H-indol-3-yl)ethanone Special details

**Table:**

Geometry. All esds (except the esd in the dihedral angle between two l.s. planes) are estimated using the full covariance matrix. The cell esds are taken into account individually in the estimation of esds in distances, angles and torsion angles; correlations between esds in cell parameters are only used when they are defined by crystal symmetry. An approximate (isotropic) treatment of cell esds is used for estimating esds involving l.s. planes.
Refinement. Refinement of F^2^ against ALL reflections. The weighted R-factor wR and goodness of fit S are based on F^2^, conventional R-factors R are based on F, with F set to zero for negative F^2^. The threshold expression of F^2^ > 2sigma(F^2^) is used only for calculating R-factors(gt) etc. and is not relevant to the choice of reflections for refinement. R-factors based on F^2^ are statistically about twice as large as those based on F, and R- factors based on ALL data will be even larger.

### (II) 2-Cyclohexyl-1-(2-phenyl-1H-indol-3-yl)ethanone Fractional atomic coordinates and isotropic or equivalent isotropic displacement parameters (Å^2^)

**Table:**

	*x*	*y*	*z*	*U*_iso_*/*U*_eq_	
C1	0.5113 (3)	0.83148 (17)	0.07278 (14)	0.0154 (5)	
C2	0.6084 (3)	0.91323 (18)	0.04474 (14)	0.0176 (5)	
H2	0.7374	0.9150	0.0468	0.021*	
C3	0.5096 (3)	0.99203 (18)	0.01371 (13)	0.0214 (6)	
H3	0.5715	1.0495	−0.0059	0.026*	
C4	0.3199 (4)	0.98840 (19)	0.01069 (15)	0.0223 (6)	
H4	0.2557	1.0431	−0.0118	0.027*	
C5	0.2234 (3)	0.90765 (19)	0.03949 (14)	0.0185 (5)	
H5	0.0944	0.9067	0.0377	0.022*	
C6	0.3208 (3)	0.82674 (17)	0.07151 (14)	0.0143 (5)	
C7	0.2707 (3)	0.73248 (17)	0.10822 (13)	0.0154 (5)	
C8	0.4327 (4)	0.68492 (16)	0.12730 (13)	0.0162 (5)	
C9	0.0841 (4)	0.70348 (16)	0.12692 (13)	0.0161 (5)	
C10	0.0489 (3)	0.62762 (16)	0.18942 (13)	0.0184 (5)	
H10A	0.1269	0.5675	0.1815	0.022*	
H10B	−0.0795	0.6054	0.1872	0.022*	
C11	0.0883 (4)	0.67346 (16)	0.26852 (13)	0.0179 (5)	
H11	0.2197	0.6927	0.2698	0.021*	
C12	0.0567 (4)	0.59550 (18)	0.33148 (14)	0.0232 (5)	
H12A	−0.0704	0.5712	0.3290	0.028*	
H12B	0.1378	0.5368	0.3232	0.028*	
C13	0.0930 (4)	0.64022 (19)	0.41026 (15)	0.0283 (6)	
H13A	0.0655	0.5890	0.4497	0.034*	
H13B	0.2231	0.6583	0.4146	0.034*	
C14	−0.0226 (4)	0.7338 (2)	0.42403 (15)	0.0314 (7)	
H14A	0.0074	0.7630	0.4745	0.038*	
H14B	−0.1527	0.7148	0.4243	0.038*	
C15	0.0112 (4)	0.81255 (19)	0.36219 (14)	0.0261 (6)	
H15A	−0.0699	0.8712	0.3706	0.031*	
H15B	0.1384	0.8366	0.3656	0.031*	
C16	−0.0229 (4)	0.76918 (18)	0.28315 (13)	0.0217 (6)	
H16A	0.0086	0.8206	0.2444	0.026*	
H16B	−0.1537	0.7533	0.2777	0.026*	
C17	0.4700 (3)	0.58519 (17)	0.16354 (13)	0.0154 (5)	
C18	0.4340 (3)	0.49591 (17)	0.12458 (13)	0.0195 (5)	
H18	0.3862	0.4983	0.0743	0.023*	
C19	0.4678 (4)	0.40310 (18)	0.15890 (15)	0.0246 (6)	
H19	0.4434	0.3422	0.1321	0.030*	
C20	0.5369 (4)	0.39947 (18)	0.23207 (15)	0.0249 (6)	
H20	0.5589	0.3360	0.2557	0.030*	
C21	0.5741 (4)	0.48777 (18)	0.27102 (15)	0.0241 (6)	
H21	0.6214	0.4850	0.3214	0.029*	
C22	0.5425 (3)	0.58032 (17)	0.23671 (14)	0.0192 (5)	
H22	0.5704	0.6409	0.2633	0.023*	
N1	0.5754 (3)	0.74414 (14)	0.10656 (11)	0.0155 (4)	
H1	0.696 (4)	0.7293 (18)	0.1088 (15)	0.019*	
O1	−0.0440 (2)	0.74797 (12)	0.09706 (9)	0.0198 (4)	

### (II) 2-Cyclohexyl-1-(2-phenyl-1H-indol-3-yl)ethanone Atomic displacement parameters (Å^2^)

**Table:**

	*U*^11^	*U*^22^	*U*^33^	*U*^12^	*U*^13^	*U*^23^
C1	0.0158 (13)	0.0173 (11)	0.0132 (12)	0.0024 (9)	−0.0004 (10)	−0.0001 (9)
C2	0.0132 (13)	0.0211 (12)	0.0185 (12)	−0.0014 (11)	0.0018 (10)	−0.0006 (10)
C3	0.0264 (15)	0.0184 (12)	0.0194 (13)	−0.0044 (11)	0.0002 (11)	0.0019 (11)
C4	0.0265 (14)	0.0192 (13)	0.0212 (15)	0.0042 (11)	−0.0032 (12)	0.0041 (12)
C5	0.0168 (13)	0.0200 (12)	0.0186 (12)	0.0017 (11)	−0.0021 (11)	0.0000 (11)
C6	0.0160 (13)	0.0160 (12)	0.0108 (12)	−0.0011 (9)	−0.0002 (10)	−0.0037 (10)
C7	0.0150 (12)	0.0182 (11)	0.0131 (12)	−0.0009 (10)	0.0002 (11)	−0.0016 (10)
C8	0.0159 (13)	0.0192 (11)	0.0135 (11)	−0.0010 (10)	0.0021 (11)	−0.0024 (9)
C9	0.0155 (12)	0.0151 (11)	0.0176 (12)	0.0026 (10)	−0.0025 (12)	−0.0056 (9)
C10	0.0134 (13)	0.0176 (11)	0.0240 (13)	−0.0012 (10)	0.0015 (11)	−0.0007 (10)
C11	0.0141 (12)	0.0201 (11)	0.0195 (12)	−0.0003 (10)	−0.0008 (12)	0.0003 (9)
C12	0.0236 (14)	0.0231 (12)	0.0229 (12)	0.0020 (12)	0.0015 (12)	0.0032 (11)
C13	0.0321 (16)	0.0327 (14)	0.0200 (13)	0.0050 (13)	0.0012 (14)	0.0049 (11)
C14	0.0389 (17)	0.0345 (15)	0.0210 (13)	0.0051 (13)	0.0030 (13)	−0.0030 (12)
C15	0.0314 (16)	0.0236 (12)	0.0234 (14)	0.0047 (11)	0.0029 (12)	−0.0040 (10)
C16	0.0247 (14)	0.0219 (12)	0.0186 (12)	0.0021 (11)	0.0019 (11)	−0.0004 (10)
C17	0.0087 (11)	0.0181 (11)	0.0194 (11)	0.0010 (10)	0.0025 (10)	0.0036 (10)
C18	0.0179 (13)	0.0211 (11)	0.0195 (12)	0.0005 (11)	0.0010 (11)	−0.0005 (10)
C19	0.0262 (14)	0.0178 (11)	0.0298 (14)	−0.0004 (11)	0.0046 (12)	−0.0015 (11)
C20	0.0238 (14)	0.0200 (12)	0.0309 (14)	0.0039 (11)	0.0020 (13)	0.0097 (11)
C21	0.0192 (13)	0.0294 (13)	0.0237 (12)	−0.0003 (12)	−0.0047 (12)	0.0089 (11)
C22	0.0172 (13)	0.0193 (11)	0.0212 (12)	−0.0031 (10)	−0.0013 (11)	0.0024 (10)
N1	0.0095 (10)	0.0176 (9)	0.0195 (10)	−0.0007 (9)	0.0004 (9)	0.0004 (8)
O1	0.0134 (9)	0.0241 (8)	0.0220 (9)	0.0012 (8)	−0.0004 (7)	−0.0007 (7)

### (II) 2-Cyclohexyl-1-(2-phenyl-1H-indol-3-yl)ethanone Geometric parameters (Å, º)

**Table:**

C1—N1	1.381 (3)	C12—H12A	0.9900
C1—C2	1.386 (3)	C12—H12B	0.9900
C1—C6	1.403 (3)	C13—C14	1.521 (4)
C2—C3	1.383 (3)	C13—H13A	0.9900
C2—H2	0.9500	C13—H13B	0.9900
C3—C4	1.398 (3)	C14—C15	1.524 (4)
C3—H3	0.9500	C14—H14A	0.9900
C4—C5	1.378 (3)	C14—H14B	0.9900
C4—H4	0.9500	C15—C16	1.522 (3)
C5—C6	1.405 (3)	C15—H15A	0.9900
C5—H5	0.9500	C15—H15B	0.9900
C6—C7	1.451 (3)	C16—H16A	0.9900
C7—C8	1.389 (3)	C16—H16B	0.9900
C7—C9	1.463 (3)	C17—C18	1.390 (3)
C8—N1	1.360 (3)	C17—C22	1.392 (3)
C8—C17	1.490 (3)	C18—C19	1.389 (3)
C9—O1	1.229 (3)	C18—H18	0.9500
C9—C10	1.509 (3)	C19—C20	1.382 (4)
C10—C11	1.542 (3)	C19—H19	0.9500
C10—H10A	0.9900	C20—C21	1.380 (3)
C10—H10B	0.9900	C20—H20	0.9500
C11—C12	1.529 (3)	C21—C22	1.384 (3)
C11—C16	1.529 (3)	C21—H21	0.9500
C11—H11	1.0000	C22—H22	0.9500
C12—C13	1.527 (3)	N1—H1	0.91 (3)
			
N1—C1—C2	129.0 (2)	C14—C13—C12	111.2 (2)
N1—C1—C6	108.1 (2)	C14—C13—H13A	109.4
C2—C1—C6	123.0 (2)	C12—C13—H13A	109.4
C3—C2—C1	117.2 (2)	C14—C13—H13B	109.4
C3—C2—H2	121.4	C12—C13—H13B	109.4
C1—C2—H2	121.4	H13A—C13—H13B	108.0
C2—C3—C4	121.0 (2)	C13—C14—C15	110.6 (2)
C2—C3—H3	119.5	C13—C14—H14A	109.5
C4—C3—H3	119.5	C15—C14—H14A	109.5
C5—C4—C3	121.8 (2)	C13—C14—H14B	109.5
C5—C4—H4	119.1	C15—C14—H14B	109.5
C3—C4—H4	119.1	H14A—C14—H14B	108.1
C4—C5—C6	118.3 (2)	C16—C15—C14	111.4 (2)
C4—C5—H5	120.9	C16—C15—H15A	109.4
C6—C5—H5	120.9	C14—C15—H15A	109.4
C1—C6—C5	118.8 (2)	C16—C15—H15B	109.4
C1—C6—C7	106.6 (2)	C14—C15—H15B	109.4
C5—C6—C7	134.6 (2)	H15A—C15—H15B	108.0
C8—C7—C6	106.1 (2)	C15—C16—C11	112.1 (2)
C8—C7—C9	129.3 (2)	C15—C16—H16A	109.2
C6—C7—C9	124.3 (2)	C11—C16—H16A	109.2
N1—C8—C7	109.72 (19)	C15—C16—H16B	109.2
N1—C8—C17	118.8 (2)	C11—C16—H16B	109.2
C7—C8—C17	131.5 (2)	H16A—C16—H16B	107.9
O1—C9—C7	119.9 (2)	C18—C17—C22	119.2 (2)
O1—C9—C10	119.8 (2)	C18—C17—C8	120.5 (2)
C7—C9—C10	119.9 (2)	C22—C17—C8	120.4 (2)
C9—C10—C11	111.12 (18)	C19—C18—C17	120.2 (2)
C9—C10—H10A	109.4	C19—C18—H18	119.9
C11—C10—H10A	109.4	C17—C18—H18	119.9
C9—C10—H10B	109.4	C20—C19—C18	119.9 (2)
C11—C10—H10B	109.4	C20—C19—H19	120.0
H10A—C10—H10B	108.0	C18—C19—H19	120.0
C12—C11—C16	110.8 (2)	C21—C20—C19	120.2 (2)
C12—C11—C10	110.88 (18)	C21—C20—H20	119.9
C16—C11—C10	112.1 (2)	C19—C20—H20	119.9
C12—C11—H11	107.6	C20—C21—C22	120.0 (2)
C16—C11—H11	107.6	C20—C21—H21	120.0
C10—C11—H11	107.6	C22—C21—H21	120.0
C13—C12—C11	111.5 (2)	C21—C22—C17	120.4 (2)
C13—C12—H12A	109.3	C21—C22—H22	119.8
C11—C12—H12A	109.3	C17—C22—H22	119.8
C13—C12—H12B	109.3	C8—N1—C1	109.46 (19)
C11—C12—H12B	109.3	C8—N1—H1	128.1 (16)
H12A—C12—H12B	108.0	C1—N1—H1	122.2 (16)
			
N1—C1—C2—C3	−179.7 (2)	C9—C10—C11—C16	56.9 (3)
C6—C1—C2—C3	−0.7 (4)	C16—C11—C12—C13	−54.2 (3)
C1—C2—C3—C4	−0.3 (4)	C10—C11—C12—C13	−179.3 (2)
C2—C3—C4—C5	1.2 (4)	C11—C12—C13—C14	56.3 (3)
C3—C4—C5—C6	−1.0 (4)	C12—C13—C14—C15	−56.8 (3)
N1—C1—C6—C5	−179.9 (2)	C13—C14—C15—C16	56.0 (3)
C2—C1—C6—C5	0.9 (4)	C14—C15—C16—C11	−54.9 (3)
N1—C1—C6—C7	1.4 (3)	C12—C11—C16—C15	53.6 (3)
C2—C1—C6—C7	−177.8 (2)	C10—C11—C16—C15	178.1 (2)
C4—C5—C6—C1	0.0 (4)	N1—C8—C17—C18	−110.2 (3)
C4—C5—C6—C7	178.2 (3)	C7—C8—C17—C18	68.9 (3)
C1—C6—C7—C8	−1.7 (3)	N1—C8—C17—C22	69.4 (3)
C5—C6—C7—C8	179.9 (3)	C7—C8—C17—C22	−111.5 (3)
C1—C6—C7—C9	172.4 (2)	C22—C17—C18—C19	0.9 (4)
C5—C6—C7—C9	−5.9 (4)	C8—C17—C18—C19	−179.5 (2)
C6—C7—C8—N1	1.4 (2)	C17—C18—C19—C20	0.2 (4)
C9—C7—C8—N1	−172.4 (2)	C18—C19—C20—C21	−0.6 (4)
C6—C7—C8—C17	−177.7 (2)	C19—C20—C21—C22	−0.1 (4)
C9—C7—C8—C17	8.5 (4)	C20—C21—C22—C17	1.2 (4)
C8—C7—C9—O1	−170.9 (2)	C18—C17—C22—C21	−1.6 (3)
C6—C7—C9—O1	16.4 (3)	C8—C17—C22—C21	178.8 (2)
C8—C7—C9—C10	16.2 (4)	C7—C8—N1—C1	−0.5 (2)
C6—C7—C9—C10	−156.5 (2)	C17—C8—N1—C1	178.7 (2)
O1—C9—C10—C11	−101.5 (2)	C2—C1—N1—C8	178.5 (2)
C7—C9—C10—C11	71.4 (3)	C6—C1—N1—C8	−0.6 (3)
C9—C10—C11—C12	−178.7 (2)		

### (II) 2-Cyclohexyl-1-(2-phenyl-1H-indol-3-yl)ethanone Hydrogen-bond geometry (Å, º)

Cg1 and Cg2 are the centroids of the N1/C1/C6–C8 ring and the C1–C6 ring, respectively.

**Table:**

*D*—H···*A*	*D*—H	H···*A*	*D*···*A*	*D*—H···*A*
N1—H1···O1^i^	0.91 (3)	1.94 (3)	2.806 (3)	158 (2)
C20—H20···*Cg*1^ii^	0.95	2.75	3.503 (3)	136
C21—H21···*Cg*2^ii^	0.95	2.61	3.437 (3)	146

Symmetry codes: (i) *x*+1, *y*, *z*; (ii) −*x*+1, *y*−1/2, −*z*+1/2.

### (III) 3,3-Dimethyl-1-(2-phenyl-1H-indol-3-yl)butan-1-one Crystal data

**Table:**

C_20_H_21_NO	*D*_x_ = 1.202 Mg m^−^^3^
*M**_r_* = 291.38	Mo *K*α radiation, λ = 0.71073 Å
Trigonal, *R*3	Cell parameters from 21024 reflections
*a* = 23.3305 (16) Å	θ = 1.7–27.5°
*c* = 15.3681 (11) Å	µ = 0.07 mm^−^^1^
*V* = 7244.3 (9) Å^3^	*T* = 100 K
*Z* = 18	Chunk, colourless
*F*(000) = 2808	0.66 × 0.60 × 0.24 mm

### (III) 3,3-Dimethyl-1-(2-phenyl-1H-indol-3-yl)butan-1-one Data collection

**Table:**

Rigaku Mercury CCD diffractometer	3070 reflections with *I* > 2σ(*I*)
Radiation source: fine-focus sealed tube	*R*_int_ = 0.037
Graphite monochromator	θ_max_ = 27.5°, θ_min_ = 1.7°
ω scans	*h* = −30→30
32188 measured reflections	*k* = −30→30
3690 independent reflections	*l* = −19→19

### (III) 3,3-Dimethyl-1-(2-phenyl-1H-indol-3-yl)butan-1-one Refinement

**Table:**

Refinement on *F*^2^	Primary atom site location: structure-invariant direct methods
Least-squares matrix: full	Secondary atom site location: difference Fourier map
*R*[*F*^2^ > 2σ(*F*^2^)] = 0.036	Hydrogen site location: inferred from neighbouring sites
*wR*(*F*^2^) = 0.092	H atoms treated by a mixture of independent and constrained refinement
*S* = 1.08	*w* = 1/[σ^2^(*F*_o_^2^) + (0.0448*P*)^2^ + 4.7609*P*] where *P* = (*F*_o_^2^ + 2*F*_c_^2^)/3
3690 reflections	(Δ/σ)_max_ < 0.001
205 parameters	Δρ_max_ = 0.28 e Å^−^^3^
0 restraints	Δρ_min_ = −0.18 e Å^−^^3^

### (III) 3,3-Dimethyl-1-(2-phenyl-1H-indol-3-yl)butan-1-one Special details

**Table:**

Geometry. All esds (except the esd in the dihedral angle between two l.s. planes) are estimated using the full covariance matrix. The cell esds are taken into account individually in the estimation of esds in distances, angles and torsion angles; correlations between esds in cell parameters are only used when they are defined by crystal symmetry. An approximate (isotropic) treatment of cell esds is used for estimating esds involving l.s. planes.
Refinement. Refinement of F^2^ against ALL reflections. The weighted R-factor wR and goodness of fit S are based on F^2^, conventional R-factors R are based on F, with F set to zero for negative F^2^. The threshold expression of F^2^ > 2sigma(F^2^) is used only for calculating R-factors(gt) etc. and is not relevant to the choice of reflections for refinement. R-factors based on F^2^ are statistically about twice as large as those based on F, and R- factors based on ALL data will be even larger.

### (III) 3,3-Dimethyl-1-(2-phenyl-1H-indol-3-yl)butan-1-one Fractional atomic coordinates and isotropic or equivalent isotropic displacement parameters (Å^2^)

**Table:**

	*x*	*y*	*z*	*U*_iso_*/*U*_eq_	
C1	0.31613 (5)	0.41861 (5)	0.13603 (6)	0.0197 (2)	
C2	0.34735 (6)	0.48614 (5)	0.15385 (7)	0.0250 (2)	
H2	0.3771	0.5181	0.1136	0.030*	
C3	0.33301 (6)	0.50441 (5)	0.23285 (7)	0.0265 (2)	
H3	0.3538	0.5500	0.2479	0.032*	
C4	0.28843 (6)	0.45692 (6)	0.29109 (7)	0.0245 (2)	
H4	0.2790	0.4710	0.3445	0.029*	
C5	0.25777 (5)	0.38987 (5)	0.27288 (7)	0.0210 (2)	
H5	0.2275	0.3582	0.3130	0.025*	
C6	0.27233 (5)	0.36965 (5)	0.19382 (6)	0.0178 (2)	
C7	0.25090 (5)	0.30630 (5)	0.15169 (6)	0.0172 (2)	
C8	0.28358 (5)	0.32044 (5)	0.07156 (6)	0.0179 (2)	
C9	0.20194 (5)	0.24318 (5)	0.18833 (6)	0.0178 (2)	
C10	0.16701 (5)	0.18172 (5)	0.13248 (7)	0.0208 (2)	
H10A	0.1919	0.1902	0.0773	0.025*	
H10B	0.1226	0.1745	0.1178	0.025*	
C11	0.15854 (5)	0.11708 (5)	0.17269 (7)	0.0232 (2)	
C12	0.10683 (6)	0.09101 (6)	0.24526 (8)	0.0325 (3)	
H12A	0.1219	0.1231	0.2931	0.049*	
H12B	0.1007	0.0487	0.2666	0.049*	
H12C	0.0647	0.0845	0.2226	0.049*	
C13	0.13580 (7)	0.06571 (6)	0.09973 (8)	0.0365 (3)	
H13A	0.0935	0.0581	0.0764	0.055*	
H13B	0.1303	0.0241	0.1228	0.055*	
H13C	0.1690	0.0820	0.0533	0.055*	
C14	0.22444 (6)	0.12844 (6)	0.20931 (9)	0.0337 (3)	
H14A	0.2392	0.1614	0.2561	0.051*	
H14B	0.2577	0.1445	0.1629	0.051*	
H14C	0.2186	0.0867	0.2324	0.051*	
C15	0.28615 (5)	0.27820 (5)	0.00135 (6)	0.0181 (2)	
C16	0.25997 (5)	0.27699 (5)	−0.08041 (7)	0.0214 (2)	
H16	0.2385	0.3019	−0.0907	0.026*	
C17	0.26503 (5)	0.23952 (5)	−0.14706 (7)	0.0234 (2)	
H17	0.2464	0.2383	−0.2025	0.028*	
C18	0.29714 (5)	0.20399 (5)	−0.13300 (7)	0.0233 (2)	
H18	0.3011	0.1789	−0.1789	0.028*	
C19	0.32356 (5)	0.20505 (5)	−0.05188 (7)	0.0252 (2)	
H19	0.3456	0.1807	−0.0421	0.030*	
C20	0.31782 (5)	0.24169 (5)	0.01498 (7)	0.0227 (2)	
H20	0.3356	0.2420	0.0707	0.027*	
N1	0.32172 (4)	0.38711 (4)	0.06303 (6)	0.02026 (19)	
H1	0.3485 (6)	0.4089 (6)	0.0173 (9)	0.024*	
O1	0.18597 (4)	0.24055 (4)	0.26571 (5)	0.02217 (17)	

### (III) 3,3-Dimethyl-1-(2-phenyl-1H-indol-3-yl)butan-1-one Atomic displacement parameters (Å^2^)

**Table:**

	*U*^11^	*U*^22^	*U*^33^	*U*^12^	*U*^13^	*U*^23^
C1	0.0212 (5)	0.0223 (5)	0.0178 (5)	0.0125 (4)	−0.0013 (4)	−0.0009 (4)
C2	0.0272 (5)	0.0206 (5)	0.0253 (5)	0.0105 (4)	0.0009 (4)	0.0013 (4)
C3	0.0311 (6)	0.0216 (5)	0.0282 (6)	0.0140 (5)	−0.0047 (5)	−0.0055 (4)
C4	0.0299 (6)	0.0291 (5)	0.0200 (5)	0.0190 (5)	−0.0036 (4)	−0.0052 (4)
C5	0.0224 (5)	0.0258 (5)	0.0180 (5)	0.0144 (4)	−0.0007 (4)	−0.0004 (4)
C6	0.0176 (4)	0.0205 (5)	0.0172 (5)	0.0111 (4)	−0.0026 (4)	−0.0002 (4)
C7	0.0179 (4)	0.0203 (5)	0.0157 (4)	0.0112 (4)	−0.0015 (4)	−0.0005 (4)
C8	0.0180 (4)	0.0199 (5)	0.0171 (5)	0.0105 (4)	−0.0023 (4)	0.0004 (4)
C9	0.0174 (4)	0.0213 (5)	0.0171 (5)	0.0115 (4)	−0.0016 (4)	0.0013 (4)
C10	0.0206 (5)	0.0209 (5)	0.0184 (5)	0.0086 (4)	−0.0014 (4)	0.0002 (4)
C11	0.0230 (5)	0.0193 (5)	0.0260 (5)	0.0095 (4)	−0.0021 (4)	−0.0005 (4)
C12	0.0329 (6)	0.0249 (6)	0.0340 (6)	0.0102 (5)	0.0048 (5)	0.0063 (5)
C13	0.0441 (7)	0.0243 (6)	0.0364 (7)	0.0135 (5)	−0.0037 (6)	−0.0070 (5)
C14	0.0314 (6)	0.0284 (6)	0.0465 (7)	0.0189 (5)	−0.0068 (5)	−0.0007 (5)
C15	0.0169 (4)	0.0177 (4)	0.0169 (5)	0.0067 (4)	0.0020 (4)	0.0007 (4)
C16	0.0234 (5)	0.0229 (5)	0.0198 (5)	0.0130 (4)	−0.0008 (4)	0.0003 (4)
C17	0.0272 (5)	0.0243 (5)	0.0170 (5)	0.0116 (4)	−0.0020 (4)	−0.0010 (4)
C18	0.0260 (5)	0.0202 (5)	0.0215 (5)	0.0099 (4)	0.0038 (4)	−0.0029 (4)
C19	0.0283 (5)	0.0252 (5)	0.0274 (6)	0.0174 (5)	−0.0003 (4)	−0.0013 (4)
C20	0.0250 (5)	0.0250 (5)	0.0199 (5)	0.0140 (4)	−0.0030 (4)	−0.0010 (4)
N1	0.0230 (4)	0.0193 (4)	0.0173 (4)	0.0098 (4)	0.0025 (3)	0.0012 (3)
O1	0.0246 (4)	0.0240 (4)	0.0166 (3)	0.0112 (3)	0.0012 (3)	0.0021 (3)

### (III) 3,3-Dimethyl-1-(2-phenyl-1H-indol-3-yl)butan-1-one Geometric parameters (Å, º)

**Table:**

C1—N1	1.3825 (13)	C11—C14	1.5308 (15)
C1—C2	1.3928 (15)	C12—H12A	0.9800
C1—C6	1.4038 (14)	C12—H12B	0.9800
C2—C3	1.3820 (16)	C12—H12C	0.9800
C2—H2	0.9500	C13—H13A	0.9800
C3—C4	1.3994 (16)	C13—H13B	0.9800
C3—H3	0.9500	C13—H13C	0.9800
C4—C5	1.3849 (15)	C14—H14A	0.9800
C4—H4	0.9500	C14—H14B	0.9800
C5—C6	1.4052 (14)	C14—H14C	0.9800
C5—H5	0.9500	C15—C16	1.3912 (14)
C6—C7	1.4541 (13)	C15—C20	1.3945 (14)
C7—C8	1.3983 (14)	C16—C17	1.3894 (15)
C7—C9	1.4520 (14)	C16—H16	0.9500
C8—N1	1.3580 (13)	C17—C18	1.3841 (15)
C8—C15	1.4827 (14)	C17—H17	0.9500
C9—O1	1.2385 (12)	C18—C19	1.3855 (15)
C9—C10	1.5128 (14)	C18—H18	0.9500
C10—C11	1.5485 (14)	C19—C20	1.3852 (15)
C10—H10A	0.9900	C19—H19	0.9500
C10—H10B	0.9900	C20—H20	0.9500
C11—C12	1.5282 (16)	N1—H1	0.909 (13)
C11—C13	1.5294 (15)		
			
N1—C1—C2	128.74 (10)	C11—C12—H12A	109.5
N1—C1—C6	107.73 (9)	C11—C12—H12B	109.5
C2—C1—C6	123.52 (9)	H12A—C12—H12B	109.5
C3—C2—C1	116.83 (10)	C11—C12—H12C	109.5
C3—C2—H2	121.6	H12A—C12—H12C	109.5
C1—C2—H2	121.6	H12B—C12—H12C	109.5
C2—C3—C4	121.09 (10)	C11—C13—H13A	109.5
C2—C3—H3	119.5	C11—C13—H13B	109.5
C4—C3—H3	119.5	H13A—C13—H13B	109.5
C5—C4—C3	121.65 (10)	C11—C13—H13C	109.5
C5—C4—H4	119.2	H13A—C13—H13C	109.5
C3—C4—H4	119.2	H13B—C13—H13C	109.5
C4—C5—C6	118.61 (10)	C11—C14—H14A	109.5
C4—C5—H5	120.7	C11—C14—H14B	109.5
C6—C5—H5	120.7	H14A—C14—H14B	109.5
C1—C6—C5	118.27 (9)	C11—C14—H14C	109.5
C1—C6—C7	106.61 (8)	H14A—C14—H14C	109.5
C5—C6—C7	135.09 (9)	H14B—C14—H14C	109.5
C8—C7—C9	129.83 (9)	C16—C15—C20	118.99 (9)
C8—C7—C6	106.41 (8)	C16—C15—C8	120.49 (9)
C9—C7—C6	123.68 (9)	C20—C15—C8	120.44 (9)
N1—C8—C7	108.84 (9)	C17—C16—C15	120.27 (10)
N1—C8—C15	118.04 (9)	C17—C16—H16	119.9
C7—C8—C15	133.04 (9)	C15—C16—H16	119.9
O1—C9—C7	119.16 (9)	C18—C17—C16	120.22 (10)
O1—C9—C10	119.45 (9)	C18—C17—H17	119.9
C7—C9—C10	121.27 (9)	C16—C17—H17	119.9
C9—C10—C11	116.27 (8)	C17—C18—C19	119.93 (9)
C9—C10—H10A	108.2	C17—C18—H18	120.0
C11—C10—H10A	108.2	C19—C18—H18	120.0
C9—C10—H10B	108.2	C20—C19—C18	119.94 (10)
C11—C10—H10B	108.2	C20—C19—H19	120.0
H10A—C10—H10B	107.4	C18—C19—H19	120.0
C12—C11—C13	109.13 (9)	C19—C20—C15	120.64 (10)
C12—C11—C14	108.98 (10)	C19—C20—H20	119.7
C13—C11—C14	109.28 (10)	C15—C20—H20	119.7
C12—C11—C10	111.70 (9)	C8—N1—C1	110.40 (8)
C13—C11—C10	107.22 (9)	C8—N1—H1	126.0 (8)
C14—C11—C10	110.49 (9)	C1—N1—H1	123.6 (8)
			
N1—C1—C2—C3	−179.69 (10)	C6—C7—C9—C10	162.05 (9)
C6—C1—C2—C3	−0.57 (16)	O1—C9—C10—C11	−46.46 (13)
C1—C2—C3—C4	−0.89 (16)	C7—C9—C10—C11	137.54 (9)
C2—C3—C4—C5	1.11 (17)	C9—C10—C11—C12	72.06 (12)
C3—C4—C5—C6	0.15 (15)	C9—C10—C11—C13	−168.44 (9)
N1—C1—C6—C5	−178.93 (9)	C9—C10—C11—C14	−49.44 (12)
C2—C1—C6—C5	1.80 (15)	N1—C8—C15—C16	−68.98 (13)
N1—C1—C6—C7	−0.50 (11)	C7—C8—C15—C16	114.61 (12)
C2—C1—C6—C7	−179.78 (9)	N1—C8—C15—C20	107.75 (11)
C4—C5—C6—C1	−1.53 (14)	C7—C8—C15—C20	−68.66 (15)
C4—C5—C6—C7	−179.39 (10)	C20—C15—C16—C17	0.45 (15)
C1—C6—C7—C8	0.81 (10)	C8—C15—C16—C17	177.23 (9)
C5—C6—C7—C8	178.84 (11)	C15—C16—C17—C18	−1.08 (16)
C1—C6—C7—C9	−176.08 (9)	C16—C17—C18—C19	0.87 (16)
C5—C6—C7—C9	1.95 (17)	C17—C18—C19—C20	−0.04 (16)
C9—C7—C8—N1	175.80 (9)	C18—C19—C20—C15	−0.59 (16)
C6—C7—C8—N1	−0.82 (11)	C16—C15—C20—C19	0.38 (15)
C9—C7—C8—C15	−7.55 (18)	C8—C15—C20—C19	−176.40 (10)
C6—C7—C8—C15	175.82 (10)	C7—C8—N1—C1	0.53 (11)
C8—C7—C9—O1	169.93 (10)	C15—C8—N1—C1	−176.69 (8)
C6—C7—C9—O1	−13.96 (14)	C2—C1—N1—C8	179.23 (10)
C8—C7—C9—C10	−14.06 (15)	C6—C1—N1—C8	0.00 (11)

### (III) 3,3-Dimethyl-1-(2-phenyl-1H-indol-3-yl)butan-1-one Hydrogen-bond geometry (Å, º)

**Table:**

*D*—H···*A*	*D*—H	H···*A*	*D*···*A*	*D*—H···*A*
N1—H1···O1^i^	0.909 (13)	1.953 (13)	2.7950 (11)	153.3 (12)

Symmetry code: (i) −*x*+*y*+1/3, −*x*+2/3, *z*−1/3.

### (IV) 3-Benzoyl-2-phenyl-1H-indole Crystal data

**Table:**

C_21_H_15_NO	*F*(000) = 1248
*M**_r_* = 297.34	*D*_x_ = 1.297 Mg m^−^^3^
Monoclinic, *P*2_1_/*c*	Mo *K*α radiation, λ = 0.71073 Å
*a* = 14.5065 (10) Å	Cell parameters from 13275 reflections
*b* = 11.7911 (9) Å	θ = 2.7–27.5°
*c* = 18.6961 (13) Å	µ = 0.08 mm^−^^1^
β = 107.782 (2)°	*T* = 100 K
*V* = 3045.1 (4) Å^3^	Lath, colourless
*Z* = 8	0.22 × 0.03 × 0.01 mm

### (IV) 3-Benzoyl-2-phenyl-1H-indole Data collection

**Table:**

Rigaku Mercury CCD diffractometer	4461 reflections with *I* > 2σ(*I*)
Radiation source: fine-focus sealed tube	*R*_int_ = 0.063
Graphite monochromator	θ_max_ = 27.5°, θ_min_ = 2.7°
ω scans	*h* = −18→18
20680 measured reflections	*k* = −15→13
6949 independent reflections	*l* = −23→24

### (IV) 3-Benzoyl-2-phenyl-1H-indole Refinement

**Table:**

Refinement on *F*^2^	Primary atom site location: structure-invariant direct methods
Least-squares matrix: full	Secondary atom site location: difference Fourier map
*R*[*F*^2^ > 2σ(*F*^2^)] = 0.076	Hydrogen site location: inferred from neighbouring sites
*wR*(*F*^2^) = 0.215	H-atom parameters constrained
*S* = 1.05	*w* = 1/[σ^2^(*F*_o_^2^) + (0.1011*P*)^2^ + 1.7166*P*] where *P* = (*F*_o_^2^ + 2*F*_c_^2^)/3
6949 reflections	(Δ/σ)_max_ < 0.001
415 parameters	Δρ_max_ = 0.58 e Å^−^^3^
0 restraints	Δρ_min_ = −0.23 e Å^−^^3^

### (IV) 3-Benzoyl-2-phenyl-1H-indole Special details

**Table:**

Geometry. All esds (except the esd in the dihedral angle between two l.s. planes) are estimated using the full covariance matrix. The cell esds are taken into account individually in the estimation of esds in distances, angles and torsion angles; correlations between esds in cell parameters are only used when they are defined by crystal symmetry. An approximate (isotropic) treatment of cell esds is used for estimating esds involving l.s. planes.
Refinement. Refinement of F^2^ against ALL reflections. The weighted R-factor wR and goodness of fit S are based on F^2^, conventional R-factors R are based on F, with F set to zero for negative F^2^. The threshold expression of F^2^ > 2sigma(F^2^) is used only for calculating R-factors(gt) etc. and is not relevant to the choice of reflections for refinement. R-factors based on F^2^ are statistically about twice as large as those based on F, and R- factors based on ALL data will be even larger.

### (IV) 3-Benzoyl-2-phenyl-1H-indole Fractional atomic coordinates and isotropic or equivalent isotropic displacement parameters (Å^2^)

**Table:**

	*x*	*y*	*z*	*U*_iso_*/*U*_eq_	
C1	0.19446 (19)	0.8013 (2)	0.39664 (13)	0.0269 (6)	
C2	0.2396 (2)	0.9067 (3)	0.40907 (14)	0.0322 (6)	
H2A	0.3052	0.9158	0.4104	0.039*	
C3	0.1852 (2)	0.9976 (3)	0.41944 (15)	0.0354 (7)	
H3	0.2133	1.0711	0.4277	0.042*	
C4	0.0883 (2)	0.9826 (3)	0.41792 (16)	0.0357 (7)	
H4	0.0525	1.0465	0.4255	0.043*	
C5	0.0442 (2)	0.8786 (3)	0.40575 (14)	0.0336 (6)	
H5	−0.0212	0.8702	0.4051	0.040*	
C6	0.09729 (19)	0.7850 (2)	0.39430 (13)	0.0281 (6)	
C7	0.07731 (18)	0.6637 (2)	0.37913 (13)	0.0264 (6)	
C8	0.16308 (17)	0.6165 (2)	0.37209 (13)	0.0263 (6)	
C9	−0.01375 (19)	0.6113 (2)	0.37465 (14)	0.0283 (6)	
C10	−0.02686 (18)	0.4863 (2)	0.36931 (14)	0.0272 (6)	
C11	0.02580 (19)	0.4146 (2)	0.42699 (14)	0.0288 (6)	
H11	0.0744	0.4450	0.4690	0.035*	
C12	0.0070 (2)	0.2997 (2)	0.42273 (15)	0.0330 (6)	
H12	0.0407	0.2513	0.4628	0.040*	
C13	−0.0617 (2)	0.2544 (3)	0.35948 (15)	0.0348 (6)	
H13	−0.0730	0.1749	0.3559	0.042*	
C14	−0.1135 (2)	0.3254 (3)	0.30192 (15)	0.0339 (6)	
H14	−0.1601	0.2945	0.2590	0.041*	
C15	−0.09743 (19)	0.4396 (3)	0.30696 (14)	0.0324 (6)	
H15	−0.1342	0.4880	0.2680	0.039*	
C16	0.18695 (18)	0.4990 (2)	0.35550 (14)	0.0276 (6)	
C17	0.26730 (19)	0.4451 (2)	0.40466 (15)	0.0302 (6)	
H17	0.3073	0.4848	0.4470	0.036*	
C18	0.2887 (2)	0.3346 (3)	0.39201 (16)	0.0353 (7)	
H18	0.3431	0.2983	0.4259	0.042*	
C19	0.2305 (2)	0.2747 (3)	0.32906 (16)	0.0350 (6)	
H19	0.2447	0.1980	0.3210	0.042*	
C20	0.1522 (2)	0.3291 (2)	0.27911 (15)	0.0317 (6)	
H20	0.1134	0.2903	0.2359	0.038*	
C21	0.13070 (19)	0.4405 (2)	0.29238 (14)	0.0292 (6)	
H21	0.0770	0.4773	0.2580	0.035*	
N1	0.23136 (15)	0.69798 (19)	0.38248 (11)	0.0284 (5)	
H1	0.2906	0.6871	0.3806	0.034*	
O1	−0.08448 (13)	0.67091 (16)	0.37534 (11)	0.0335 (5)	
C22	0.69538 (18)	0.5190 (2)	0.40301 (13)	0.0260 (6)	
C23	0.74041 (19)	0.4145 (2)	0.41931 (14)	0.0305 (6)	
H23	0.8060	0.4039	0.4210	0.037*	
C24	0.6850 (2)	0.3256 (2)	0.43313 (15)	0.0338 (6)	
H24	0.7126	0.2521	0.4438	0.041*	
C25	0.5884 (2)	0.3439 (3)	0.43146 (15)	0.0348 (7)	
H25	0.5524	0.2825	0.4422	0.042*	
C26	0.54494 (19)	0.4484 (2)	0.41476 (14)	0.0314 (6)	
H26	0.4797	0.4591	0.4139	0.038*	
C27	0.59814 (18)	0.5380 (2)	0.39919 (13)	0.0265 (6)	
C28	0.57881 (18)	0.6581 (2)	0.38018 (13)	0.0270 (6)	
C29	0.66528 (18)	0.7025 (2)	0.37257 (13)	0.0265 (6)	
C30	0.48733 (19)	0.7126 (2)	0.37205 (14)	0.0292 (6)	
C31	0.47679 (19)	0.8381 (2)	0.36448 (14)	0.0280 (6)	
C32	0.53489 (19)	0.9104 (2)	0.41874 (14)	0.0300 (6)	
H32	0.5833	0.8801	0.4608	0.036*	
C33	0.5216 (2)	1.0267 (3)	0.41100 (15)	0.0345 (6)	
H33	0.5605	1.0763	0.4482	0.041*	
C34	0.4517 (2)	1.0708 (3)	0.34902 (16)	0.0362 (7)	
H34	0.4439	1.1507	0.3435	0.043*	
C35	0.3934 (2)	1.0000 (3)	0.29536 (15)	0.0369 (7)	
H35	0.3454	1.0311	0.2533	0.044*	
C36	0.40478 (19)	0.8845 (3)	0.30272 (14)	0.0320 (6)	
H36	0.3640	0.8357	0.2661	0.038*	
C37	0.68882 (19)	0.8182 (2)	0.35194 (14)	0.0272 (6)	
C38	0.76544 (19)	0.8797 (2)	0.40043 (15)	0.0305 (6)	
H38	0.8052	0.8450	0.4451	0.037*	
C39	0.7836 (2)	0.9894 (3)	0.38400 (16)	0.0355 (7)	
H39	0.8355	1.0304	0.4174	0.043*	
C40	0.7257 (2)	1.0408 (3)	0.31815 (16)	0.0338 (6)	
H40	0.7375	1.1171	0.3072	0.041*	
C41	0.65150 (19)	0.9803 (2)	0.26904 (14)	0.0302 (6)	
H41	0.6129	1.0146	0.2238	0.036*	
C42	0.63344 (19)	0.8698 (2)	0.28572 (14)	0.0285 (6)	
H42	0.5826	0.8285	0.2515	0.034*	
N2	0.73368 (16)	0.62092 (19)	0.38683 (11)	0.0287 (5)	
H2	0.7935	0.6305	0.3860	0.034*	
O2	0.41519 (13)	0.65488 (17)	0.37109 (11)	0.0336 (5)	

### (IV) 3-Benzoyl-2-phenyl-1H-indole Atomic displacement parameters (Å^2^)

**Table:**

	*U*^11^	*U*^22^	*U*^33^	*U*^12^	*U*^13^	*U*^23^
C1	0.0263 (13)	0.0337 (15)	0.0227 (11)	0.0053 (11)	0.0106 (10)	0.0020 (10)
C2	0.0239 (13)	0.0450 (18)	0.0305 (13)	−0.0039 (12)	0.0124 (11)	0.0017 (12)
C3	0.0397 (16)	0.0324 (16)	0.0347 (14)	−0.0063 (13)	0.0123 (12)	0.0000 (12)
C4	0.0336 (15)	0.0356 (17)	0.0384 (15)	0.0078 (13)	0.0120 (12)	−0.0016 (12)
C5	0.0233 (13)	0.0487 (18)	0.0316 (13)	0.0019 (13)	0.0128 (11)	−0.0007 (12)
C6	0.0260 (13)	0.0363 (16)	0.0236 (11)	−0.0027 (11)	0.0101 (10)	0.0014 (11)
C7	0.0181 (12)	0.0379 (16)	0.0252 (11)	0.0038 (11)	0.0096 (9)	0.0005 (11)
C8	0.0192 (12)	0.0378 (16)	0.0245 (11)	0.0006 (11)	0.0105 (9)	0.0036 (11)
C9	0.0235 (13)	0.0380 (16)	0.0265 (12)	0.0033 (11)	0.0123 (10)	0.0019 (11)
C10	0.0238 (12)	0.0339 (15)	0.0284 (12)	0.0008 (11)	0.0148 (10)	0.0000 (11)
C11	0.0244 (13)	0.0380 (17)	0.0260 (12)	0.0010 (12)	0.0104 (10)	0.0009 (11)
C12	0.0257 (14)	0.0383 (17)	0.0352 (14)	0.0039 (12)	0.0096 (11)	0.0055 (12)
C13	0.0295 (14)	0.0391 (17)	0.0400 (15)	−0.0048 (13)	0.0169 (12)	−0.0035 (12)
C14	0.0263 (14)	0.0468 (19)	0.0306 (13)	−0.0038 (13)	0.0116 (11)	−0.0048 (12)
C15	0.0241 (13)	0.0497 (19)	0.0252 (12)	0.0022 (12)	0.0101 (10)	0.0047 (12)
C16	0.0250 (13)	0.0365 (16)	0.0275 (12)	−0.0026 (11)	0.0170 (10)	0.0010 (11)
C17	0.0248 (13)	0.0389 (17)	0.0317 (13)	−0.0036 (12)	0.0156 (11)	−0.0023 (11)
C18	0.0268 (14)	0.0488 (19)	0.0353 (14)	0.0055 (13)	0.0170 (11)	0.0076 (13)
C19	0.0348 (15)	0.0346 (16)	0.0443 (15)	0.0022 (13)	0.0251 (13)	−0.0004 (12)
C20	0.0293 (14)	0.0382 (16)	0.0329 (13)	−0.0057 (12)	0.0175 (11)	−0.0067 (12)
C21	0.0247 (13)	0.0399 (17)	0.0265 (12)	−0.0019 (12)	0.0129 (10)	−0.0017 (11)
N1	0.0192 (10)	0.0386 (14)	0.0305 (11)	−0.0009 (10)	0.0121 (9)	−0.0011 (9)
O1	0.0230 (10)	0.0385 (12)	0.0432 (11)	0.0058 (8)	0.0162 (8)	0.0060 (9)
C22	0.0252 (13)	0.0331 (15)	0.0215 (11)	−0.0023 (11)	0.0096 (10)	−0.0008 (10)
C23	0.0219 (12)	0.0462 (18)	0.0264 (12)	0.0042 (12)	0.0118 (10)	−0.0026 (11)
C24	0.0398 (16)	0.0326 (16)	0.0295 (13)	0.0087 (13)	0.0115 (12)	0.0023 (11)
C25	0.0361 (16)	0.0402 (18)	0.0312 (13)	−0.0105 (13)	0.0147 (12)	−0.0013 (12)
C26	0.0234 (13)	0.0436 (17)	0.0289 (13)	−0.0018 (12)	0.0108 (11)	−0.0012 (12)
C27	0.0239 (13)	0.0342 (15)	0.0228 (11)	0.0009 (11)	0.0091 (10)	−0.0029 (10)
C28	0.0202 (12)	0.0373 (16)	0.0259 (12)	−0.0029 (11)	0.0107 (10)	−0.0005 (11)
C29	0.0188 (12)	0.0387 (16)	0.0240 (11)	0.0022 (11)	0.0093 (9)	−0.0042 (11)
C30	0.0225 (13)	0.0417 (17)	0.0269 (12)	−0.0016 (12)	0.0126 (10)	−0.0051 (11)
C31	0.0239 (13)	0.0360 (16)	0.0282 (12)	0.0006 (11)	0.0139 (10)	−0.0010 (11)
C32	0.0277 (13)	0.0394 (17)	0.0259 (12)	−0.0013 (12)	0.0126 (10)	−0.0014 (11)
C33	0.0341 (15)	0.0402 (17)	0.0322 (14)	0.0004 (13)	0.0147 (12)	−0.0032 (12)
C34	0.0384 (16)	0.0377 (17)	0.0390 (15)	0.0076 (13)	0.0215 (13)	0.0036 (13)
C35	0.0303 (15)	0.052 (2)	0.0316 (14)	0.0102 (14)	0.0140 (12)	0.0078 (13)
C36	0.0227 (13)	0.0485 (18)	0.0268 (12)	−0.0007 (12)	0.0107 (10)	−0.0022 (12)
C37	0.0253 (13)	0.0325 (15)	0.0288 (12)	0.0013 (11)	0.0159 (10)	−0.0015 (11)
C38	0.0218 (13)	0.0395 (17)	0.0330 (13)	0.0042 (12)	0.0123 (10)	0.0019 (12)
C39	0.0246 (13)	0.0461 (19)	0.0391 (15)	−0.0081 (13)	0.0144 (12)	−0.0036 (13)
C40	0.0326 (15)	0.0347 (16)	0.0402 (15)	−0.0040 (12)	0.0200 (12)	0.0028 (12)
C41	0.0265 (13)	0.0398 (16)	0.0290 (12)	0.0042 (12)	0.0156 (11)	0.0065 (11)
C42	0.0251 (13)	0.0377 (16)	0.0271 (12)	0.0008 (11)	0.0145 (10)	−0.0023 (11)
N2	0.0208 (11)	0.0379 (14)	0.0304 (11)	0.0027 (10)	0.0122 (9)	0.0013 (10)
O2	0.0197 (9)	0.0404 (12)	0.0443 (11)	−0.0034 (8)	0.0151 (8)	−0.0045 (9)

### (IV) 3-Benzoyl-2-phenyl-1H-indole Geometric parameters (Å, º)

**Table:**

C1—N1	1.389 (3)	C22—C23	1.385 (4)
C1—C2	1.391 (4)	C22—N2	1.395 (3)
C1—C6	1.410 (4)	C22—C27	1.408 (3)
C2—C3	1.379 (4)	C23—C24	1.392 (4)
C2—H2A	0.9500	C23—H23	0.9500
C3—C4	1.408 (4)	C24—C25	1.409 (4)
C3—H3	0.9500	C24—H24	0.9500
C4—C5	1.370 (4)	C25—C26	1.375 (4)
C4—H4	0.9500	C25—H25	0.9500
C5—C6	1.399 (4)	C26—C27	1.391 (4)
C5—H5	0.9500	C26—H26	0.9500
C6—C7	1.470 (4)	C27—C28	1.466 (4)
C7—C8	1.405 (3)	C28—C29	1.406 (3)
C7—C9	1.437 (4)	C28—C30	1.440 (4)
C8—N1	1.351 (3)	C29—N2	1.348 (3)
C8—C16	1.484 (4)	C29—C37	1.487 (4)
C9—O1	1.247 (3)	C30—O2	1.244 (3)
C9—C10	1.486 (4)	C30—C31	1.491 (4)
C10—C11	1.399 (4)	C31—C32	1.394 (4)
C10—C15	1.408 (4)	C31—C36	1.410 (4)
C11—C12	1.379 (4)	C32—C33	1.386 (4)
C11—H11	0.9500	C32—H32	0.9500
C12—C13	1.399 (4)	C33—C34	1.387 (4)
C12—H12	0.9500	C33—H33	0.9500
C13—C14	1.389 (4)	C34—C35	1.379 (4)
C13—H13	0.9500	C34—H34	0.9500
C14—C15	1.364 (4)	C35—C36	1.374 (4)
C14—H14	0.9500	C35—H35	0.9500
C15—H15	0.9500	C36—H36	0.9500
C16—C21	1.395 (4)	C37—C42	1.393 (4)
C16—C17	1.398 (4)	C37—C38	1.402 (4)
C17—C18	1.377 (4)	C38—C39	1.374 (4)
C17—H17	0.9500	C38—H38	0.9500
C18—C19	1.411 (4)	C39—C40	1.398 (4)
C18—H18	0.9500	C39—H39	0.9500
C19—C20	1.387 (4)	C40—C41	1.381 (4)
C19—H19	0.9500	C40—H40	0.9500
C20—C21	1.389 (4)	C41—C42	1.384 (4)
C20—H20	0.9500	C41—H41	0.9500
C21—H21	0.9500	C42—H42	0.9500
N1—H1	0.8800	N2—H2	0.8800
			
N1—C1—C2	128.8 (2)	C23—C22—N2	128.5 (2)
N1—C1—C6	108.3 (2)	C23—C22—C27	123.4 (2)
C2—C1—C6	122.9 (2)	N2—C22—C27	108.1 (2)
C3—C2—C1	117.2 (2)	C22—C23—C24	116.8 (2)
C3—C2—H2A	121.4	C22—C23—H23	121.6
C1—C2—H2A	121.4	C24—C23—H23	121.6
C2—C3—C4	120.7 (3)	C23—C24—C25	120.5 (3)
C2—C3—H3	119.7	C23—C24—H24	119.8
C4—C3—H3	119.7	C25—C24—H24	119.8
C5—C4—C3	121.9 (3)	C26—C25—C24	121.8 (3)
C5—C4—H4	119.1	C26—C25—H25	119.1
C3—C4—H4	119.1	C24—C25—H25	119.1
C4—C5—C6	118.7 (2)	C25—C26—C27	118.8 (2)
C4—C5—H5	120.6	C25—C26—H26	120.6
C6—C5—H5	120.6	C27—C26—H26	120.6
C5—C6—C1	118.6 (3)	C26—C27—C22	118.7 (2)
C5—C6—C7	135.4 (2)	C26—C27—C28	135.0 (2)
C1—C6—C7	106.0 (2)	C22—C27—C28	106.3 (2)
C8—C7—C9	130.5 (3)	C29—C28—C30	130.2 (3)
C8—C7—C6	106.0 (2)	C29—C28—C27	105.9 (2)
C9—C7—C6	123.5 (2)	C30—C28—C27	124.0 (2)
N1—C8—C7	109.7 (2)	N2—C29—C28	109.9 (2)
N1—C8—C16	119.1 (2)	N2—C29—C37	119.5 (2)
C7—C8—C16	131.2 (2)	C28—C29—C37	130.6 (2)
O1—C9—C7	120.1 (3)	O2—C30—C28	120.1 (3)
O1—C9—C10	118.1 (2)	O2—C30—C31	118.7 (2)
C7—C9—C10	121.8 (2)	C28—C30—C31	121.2 (2)
C11—C10—C15	119.3 (3)	C32—C31—C36	119.4 (3)
C11—C10—C9	121.3 (2)	C32—C31—C30	121.1 (2)
C15—C10—C9	119.3 (2)	C36—C31—C30	119.5 (2)
C12—C11—C10	119.8 (3)	C33—C32—C31	119.7 (3)
C12—C11—H11	120.1	C33—C32—H32	120.2
C10—C11—H11	120.1	C31—C32—H32	120.2
C11—C12—C13	120.1 (3)	C32—C33—C34	120.1 (3)
C11—C12—H12	119.9	C32—C33—H33	120.0
C13—C12—H12	120.0	C34—C33—H33	120.0
C14—C13—C12	120.1 (3)	C35—C34—C33	120.7 (3)
C14—C13—H13	120.0	C35—C34—H34	119.7
C12—C13—H13	120.0	C33—C34—H34	119.7
C15—C14—C13	120.1 (3)	C36—C35—C34	120.0 (3)
C15—C14—H14	120.0	C36—C35—H35	120.0
C13—C14—H14	120.0	C34—C35—H35	120.0
C14—C15—C10	120.6 (3)	C35—C36—C31	120.2 (3)
C14—C15—H15	119.7	C35—C36—H36	119.9
C10—C15—H15	119.7	C31—C36—H36	119.9
C21—C16—C17	119.1 (3)	C42—C37—C38	118.5 (3)
C21—C16—C8	121.7 (2)	C42—C37—C29	121.0 (2)
C17—C16—C8	119.2 (2)	C38—C37—C29	120.4 (2)
C18—C17—C16	120.3 (3)	C39—C38—C37	120.7 (3)
C18—C17—H17	119.9	C39—C38—H38	119.7
C16—C17—H17	119.9	C37—C38—H38	119.7
C17—C18—C19	120.5 (3)	C38—C39—C40	120.0 (3)
C17—C18—H18	119.7	C38—C39—H39	120.0
C19—C18—H18	119.7	C40—C39—H39	120.0
C20—C19—C18	119.2 (3)	C41—C40—C39	119.9 (3)
C20—C19—H19	120.4	C41—C40—H40	120.1
C18—C19—H19	120.4	C39—C40—H40	120.1
C21—C20—C19	120.0 (3)	C42—C41—C40	119.9 (3)
C21—C20—H20	120.0	C42—C41—H41	120.0
C19—C20—H20	120.0	C40—C41—H41	120.0
C20—C21—C16	120.9 (3)	C41—C42—C37	120.9 (3)
C20—C21—H21	119.6	C41—C42—H42	119.5
C16—C21—H21	119.6	C37—C42—H42	119.5
C8—N1—C1	110.1 (2)	C29—N2—C22	109.8 (2)
C8—N1—H1	125.0	C29—N2—H2	125.1
C1—N1—H1	125.0	C22—N2—H2	125.1
			
N1—C1—C2—C3	177.9 (2)	N2—C22—C23—C24	−179.7 (2)
C6—C1—C2—C3	0.1 (4)	C27—C22—C23—C24	−0.7 (4)
C1—C2—C3—C4	0.5 (4)	C22—C23—C24—C25	−1.0 (4)
C2—C3—C4—C5	−0.4 (4)	C23—C24—C25—C26	1.4 (4)
C3—C4—C5—C6	−0.2 (4)	C24—C25—C26—C27	0.0 (4)
C4—C5—C6—C1	0.7 (4)	C25—C26—C27—C22	−1.6 (4)
C4—C5—C6—C7	−179.2 (3)	C25—C26—C27—C28	−179.4 (3)
N1—C1—C6—C5	−178.9 (2)	C23—C22—C27—C26	2.0 (4)
C2—C1—C6—C5	−0.7 (4)	N2—C22—C27—C26	−178.8 (2)
N1—C1—C6—C7	1.1 (3)	C23—C22—C27—C28	−179.6 (2)
C2—C1—C6—C7	179.3 (2)	N2—C22—C27—C28	−0.4 (3)
C5—C6—C7—C8	178.9 (3)	C26—C27—C28—C29	179.0 (3)
C1—C6—C7—C8	−1.1 (3)	C22—C27—C28—C29	1.0 (3)
C5—C6—C7—C9	−2.0 (4)	C26—C27—C28—C30	−1.0 (4)
C1—C6—C7—C9	178.1 (2)	C22—C27—C28—C30	−179.0 (2)
C9—C7—C8—N1	−178.4 (2)	C30—C28—C29—N2	178.7 (2)
C6—C7—C8—N1	0.7 (3)	C27—C28—C29—N2	−1.3 (3)
C9—C7—C8—C16	2.2 (5)	C30—C28—C29—C37	−1.8 (4)
C6—C7—C8—C16	−178.8 (2)	C27—C28—C29—C37	178.2 (2)
C8—C7—C9—O1	−173.0 (2)	C29—C28—C30—O2	170.2 (2)
C6—C7—C9—O1	8.1 (4)	C27—C28—C30—O2	−9.8 (4)
C8—C7—C9—C10	7.1 (4)	C29—C28—C30—C31	−9.7 (4)
C6—C7—C9—C10	−171.7 (2)	C27—C28—C30—C31	170.4 (2)
O1—C9—C10—C11	−117.3 (3)	O2—C30—C31—C32	123.6 (3)
C7—C9—C10—C11	62.6 (3)	C28—C30—C31—C32	−56.5 (3)
O1—C9—C10—C15	59.2 (3)	O2—C30—C31—C36	−54.3 (3)
C7—C9—C10—C15	−121.0 (3)	C28—C30—C31—C36	125.6 (3)
C15—C10—C11—C12	−1.0 (4)	C36—C31—C32—C33	−0.5 (4)
C9—C10—C11—C12	175.4 (2)	C30—C31—C32—C33	−178.4 (2)
C10—C11—C12—C13	2.7 (4)	C31—C32—C33—C34	−0.9 (4)
C11—C12—C13—C14	−2.1 (4)	C32—C33—C34—C35	1.4 (4)
C12—C13—C14—C15	0.0 (4)	C33—C34—C35—C36	−0.5 (4)
C13—C14—C15—C10	1.6 (4)	C34—C35—C36—C31	−0.8 (4)
C11—C10—C15—C14	−1.1 (4)	C32—C31—C36—C35	1.3 (4)
C9—C10—C15—C14	−177.6 (2)	C30—C31—C36—C35	179.3 (2)
N1—C8—C16—C21	−125.5 (3)	N2—C29—C37—C42	124.5 (3)
C7—C8—C16—C21	53.9 (4)	C28—C29—C37—C42	−55.0 (4)
N1—C8—C16—C17	55.1 (3)	N2—C29—C37—C38	−57.9 (3)
C7—C8—C16—C17	−125.5 (3)	C28—C29—C37—C38	122.6 (3)
C21—C16—C17—C18	−1.9 (4)	C42—C37—C38—C39	1.9 (4)
C8—C16—C17—C18	177.5 (2)	C29—C37—C38—C39	−175.7 (2)
C16—C17—C18—C19	0.5 (4)	C37—C38—C39—C40	−0.4 (4)
C17—C18—C19—C20	1.2 (4)	C38—C39—C40—C41	−1.2 (4)
C18—C19—C20—C21	−1.5 (4)	C39—C40—C41—C42	1.1 (4)
C19—C20—C21—C16	0.1 (4)	C40—C41—C42—C37	0.4 (4)
C17—C16—C21—C20	1.6 (4)	C38—C37—C42—C41	−1.9 (4)
C8—C16—C21—C20	−177.8 (2)	C29—C37—C42—C41	175.7 (2)
C7—C8—N1—C1	0.0 (3)	C28—C29—N2—C22	1.1 (3)
C16—C8—N1—C1	179.5 (2)	C37—C29—N2—C22	−178.5 (2)
C2—C1—N1—C8	−178.8 (2)	C23—C22—N2—C29	178.7 (2)
C6—C1—N1—C8	−0.7 (3)	C27—C22—N2—C29	−0.4 (3)

### (IV) 3-Benzoyl-2-phenyl-1H-indole Hydrogen-bond geometry (Å, º)

Cg8, Cg1, Cg7, Cg3 and Cg6 are the centroids of the C31–C36, N1/C1/C6–C8, C22–C27, C10–C15 and N2/C22/C27–C29 rings, respectively.

**Table:**

*D*—H···*A*	*D*—H	H···*A*	*D*···*A*	*D*—H···*A*
N1—H1···O2	0.88	1.91	2.786 (3)	176
N2—H2···O1^i^	0.88	1.90	2.775 (3)	171
C20—H20···O1^ii^	0.95	2.44	3.324 (3)	155
C41—H41···O2^iii^	0.95	2.37	3.239 (3)	152
C2—H2*A*···*Cg*8	0.95	2.81	3.715 (3)	158
C14—H14···*Cg*1^ii^	0.95	2.89	3.616 (3)	134
C17—H17···*Cg*7^iv^	0.95	2.62	3.508 (3)	156
C23—H23···*Cg*3^i^	0.95	2.72	3.608 (3)	156
C35—H35···*Cg*6^iii^	0.95	2.80	3.527 (3)	134

Symmetry codes: (i) *x*+1, *y*, *z*; (ii) −*x*, *y*−1/2, −*z*+1/2; (iii) −*x*+1, *y*+1/2, −*z*+1/2; (iv) −*x*+1, −*y*+1, −*z*+1.
